# Laser‐Induced Photothermal Conversion to Hemispherical MoS_2_ Enabling Non‐Contact Self‐Powering Image Sensor

**DOI:** 10.1002/advs.202513887

**Published:** 2025-09-08

**Authors:** Chan‐Jin Kim, Kwang‐Hun Choi, Taegyeong Lim, Seokjun Cha, Yoon‐ho Lee, Da Som Song, Ye Jun Lee, Sunhwa Hong, Jun Ho Kim, Seung Hoon Lee, Saewon Kang, Wooseok Song, Sung Myung, Jongsun Lim, Sun Sook Lee, Ki‐Seok Ahn, Soonmin Yim, Byung Hee Hong, Sungwoong Park

**Affiliations:** ^1^ Graphene Research Center Advanced Institute of Convergence Technology & Department of Chemistry College of Natural Sciences Seoul National University Seoul 08826 Republic of Korea; ^2^ Department of Materials Science and Engineering Korea Advanced Institute of Science and Technology (KAIST) Daejeon 34141 Republic of Korea; ^3^ Department of Mechanical Engineering Seoul National University 1 Gwanak‐ro Gwanak‐gu Seoul 08826 Republic of Korea; ^4^ Department of Materials Science and Engineering Research Institute of Advanced Materials 1 Gwanak‐ro, Gwanak‐gu Seoul 08826 Republic of Korea; ^5^ Thin Film Materials Research Center Korea Research Institute of Chemical Technology Daejeon 34114 Republic of Korea

**Keywords:** energy harvesting, hemisphere, image sensor, molybdenum disulfide, non‐contact mode

## Abstract

Molybdenum disulfide (MoS_2_) has recently emerged as a promising material for the development of triboelectric nanogenerators (TENGs) owing to its inherently negative triboelectric properties when paired with polymeric layers, along with its notable transparency and mechanical flexibility. However, MoS_2_‐based TENGs operating in the contact‐separation mode encounter critical limitations, including mechanical wear and limited triboelectric performance, particularly within the constraints of conventional 2D geometries. This paper reports the novel one‐step laser‐assisted synthesis of hemispherical MoS_2_ through the controlled nucleation and growth of MoS_2_ precursor seeds. The hemispherical structures synthesized at the optimized precursor concentration (0.32 m) exhibit a mean diameter of 234.4 nm with a standard deviation of 30.4 nm, uniformly distributed across a wafer‐scale substrate. Hemispherical MoS_2_ significantly increases the electric‐field concentration, making it well‐suited for integration into noncontact‐mode TENG (NC‐TENG) devices. Compared with a flat MoS_2_‐based NC‐TENG, a hemispherical MoS_2_‐based NC‐TENG demonstrates a 22‐fold increase in average capacitance (≈112 pF, D_vertical_ = 2 mm) and a 37‐fold increase in open‐circuit voltage (≈2.25 V, D_vertical_ = 2 mm), while extending the operational distance to 10 mm. Furthermore, this advanced hemispherical MoS_2_ architecture is employed to fabricate a self‐powered image sensor array, underscoring its potential for broader applications.

## Graphical abstract

The laser‐assisted method allows the direct synthesis of hemispherical MoS_2_ (*h*‐MoS_2_). Through curvature‐induced electric‐field amplification, the *h*‐MoS_2_‐based noncontact TENG exhibited a 37‐fold increase in voltage output and operated stably at distances of up to 10 mm. The integrated sensor array based on pixelated *h*‐MoS_2_ enables the shape‐specific, contactless detection of external stimuli, offering a scalable route toward noncontact electronic systems.

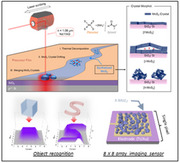

## 1 Introduction

Triboelectric nanogenerator (TENG) technology has emerged as a sustainable approach for energy harvesting and self‐powered devices by converting mechanical energy into electrical signals, enabling significant advances in smart wearable devices, motion monitoring systems, and tactile sensors.^[^ ^1^, ^2^, ^3^, ^4^ ^]^ Realizing these various applications requires triboelectric materials with tunable mechanical and electrical properties, high chemical stability, and compatibility with miniaturized or wearable platforms, which are challenging to achieve using conventional bulk materials. In this context, 2D materials have been regarded as promising candidates due to their unique electrical, mechanical, and chemical properties.^[^ ^5^, ^6^, ^7^, ^8^, ^9^, ^10^ ^]^ In particular, molybdenum disulfide (MoS_2_) has attracted considerable attention for triboelectric nanogenerator (TENG) applications owing to its outstanding mechanical flexibility and stable chemical structure, as well as its intrinsically negative triboelectric nature, which enables strong charge interactions with positive triboelectric materials such as polyethylene terephthalate (PET) and nylon.^[^ ^11^, ^12^, ^13^ ^]^ These properties have driven notable advancements in energy‐conversion efficiency while enabling the miniaturization of wearable Internet of Things (IoT) devices.^[^ ^14^, ^15^ ^]^ However, conventional TENGs based on MoS_2_ with a flat morphology exhibit relatively low triboelectric performance and limited effective surface area, restricting their utilization primarily to contact‐mode energy harvesting and 2D spatial sensing.^[^ ^16^, ^17^ ^]^ Moreover, the widely adopted contact‐separation mode in TENG systems suffers from performance degradation over time due to mechanical wear of the friction layer, further hindering long‐term or high‐frequency applications.^[^ ^4^, ^5^, ^6^ ^]^ These constraints significantly limit their potential in advanced sustainable energy harvesting and 3D sensing devices operating in the noncontact TENG mode, which require strong electrostatic induction at an extended working distance.^[^ ^18^ ^]^

To address these limitations, research on MoS_2_‐based TENGs has focused on developing MoS_2_‐polymer composites, in which MoS_2_ functions as a charge‐trapping site or dielectric‐modifying component to increase the triboelectric charge densities.^[^ ^19^, ^20^ ^]^ In contrast, efforts to enhance the intrinsic triboelectric output of MoS_2_ itself have been relatively limited. For instance, applying external strain to modulate the lattice spacing of MoS_2_ was shown to adjust its work function and increase the electron‐transfer efficiency.^[^ ^21^ ^]^ In addition, surface modification of MoS_2_ with aromatic carboxylic acids was employed to modify its work function and surface charge density, improving the triboelectric output.^[^ ^16^ ^]^ Although these strain and surface engineering approaches have incrementally enhanced the intrinsic triboelectric properties of MoS_2_, it is still necessary to overcome the fundamental limitations of its triboelectric response arising from its planar and lamellar structures.^[^ ^17^ ^]^ In particular, the difficulty in modulating the MoS_2_ structure without sacrificing crystallinity or introducing additional fabrication complexity has hindered the development of morphology‐engineered MoS_2_ structures for triboelectric performance.

Herein, we propose a novel one‐step laser‐assisted synthesis platform for fabricating hemispherical MoS_2_ crystals by precisely controlling the concentration of a tetrathiomolybdate ((NH_4_)_2_MoS_4_) precursor solution. Compared with conventional chemical vapor deposition methods, this approach offers significant advantages, including reduced synthesis time, enhanced structural control, and minimal thermal impact on the substrate. The resulting hemispherical MoS_2_ crystals exhibited a remarkable 22‐fold increase in the average capacitance (≈112 pF, D = 2 mm) compared with planar MoS_2_ structures, leading to a 37‐fold enhancement in the open‐circuit voltage (OCV) when implemented in self‐powering devices. Furthermore, we demonstrated the integration of hemispherical MoS_2_ crystals into a noncontact self‐powered image sensor array capable of detecting 3D objects, highlighting the potential of this platform for advanced multidimensional sensing applications.

## 2 Results and Discussion

**Figure 1:**
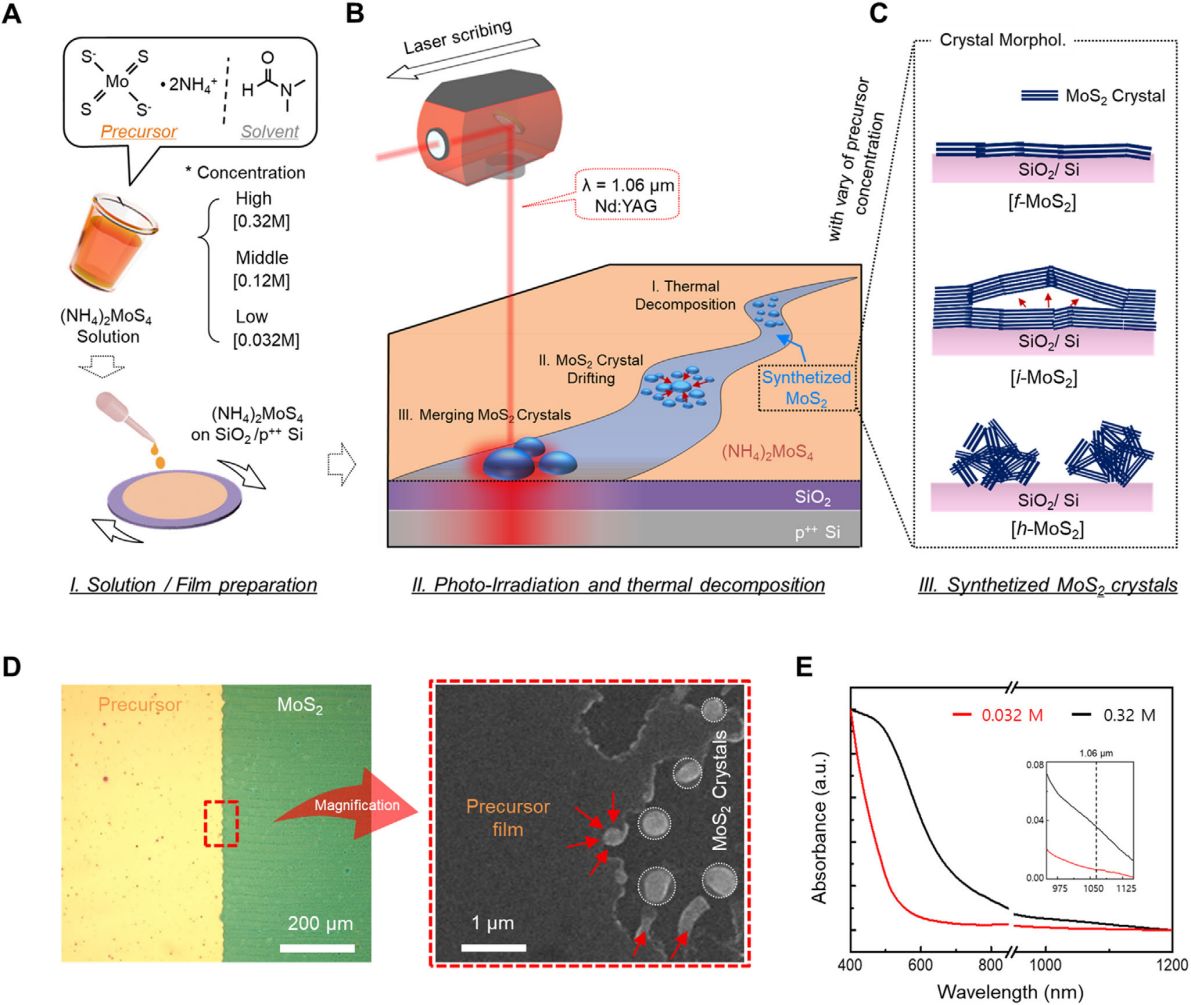
A) Schematic of the precursor preparation and film deposition. Precursor solutions with three different concentrations—low (0.032 m), medium (0.12 m), and high (0.32 m)—were spin‐coated onto an SiO_2_/p^++^ Si wafer. B) Schematic of laser‐induced photothermal decomposition of the precursor film into MoS_2_ crystals. C) Cross‐sectional illustration of the resulting MoS_2_ morphologies: *f*‐MoS_2_, *i*‐MoS_2_, and *h*‐MoS_2_. D) OM (left) and SEM (right) images of the laser‐annealed 0.32 m precursor film. The SEM image highlights the nucleation behavior of (NH_4_)_2_MoS_4_ at the interface between the precursor region and the crystallized *h*‐MoS_2_. E) UV–vis spectra of 0.032 m and 0.32 m precursor solutions. The inset shows the absorbance difference at 1.06 µm, indicating increased light absorption in the highly concentrated precursor.

Hemispherical MoS_2_ was synthesized using a laser‐assisted thermal annealing technique, initiated by the preparation and deposition of an MoS_2_ precursor solution (**Figure** **1**A). (NH_4_)_2_MoS_4_ was dissolved in a mixed organic solvent at various concentrations, and the precursor concentration was adjusted to three levels: low (0.032 m), medium (0.12 m), and high (0.32 m). These concentrations corresponded to the formation of flat, intermediate, and hemispherical MoS_2_ structures, respectively, upon subsequent annealing. A uniform precursor film was spin‐coated onto a 1.5 cm × 1.5 cm SiO_2_/p^++^ Si wafer, and a photo‐irradiation process was performed using a pulsed fiber laser (λ ≈ 1.06 µm) at an optimal laser fluence of 2.6 J/cm^2^, sufficient to thermolyze (NH_4_)_2_MoS_4_ seeds while avoiding the formation of wrinkled structures caused by excessive photothermal energy (Figure S1, Supporting Information). Consequently, the localized photothermal energy drives rapid photothermal activation, decomposing the precursor film and converting it into the hemispherical MoS_2_ crystals.^[^ ^22^ ^]^ The surficial drifting behavior of the MoS_2_ seeds was strongly dependent on the precursor concentration during this laser‐induced thermolysis step (Figure 1B). Scanning electron microscopy (SEM) images (Figure S2, Supporting Information) indicated that the 0.032 m precursor yielded an atomically thin, flat MoS_2_ film. At 0.12 m, crystallization followed the surficial drifting pathways of the precursor seeds, leading to irregular, partially elevated domains. In contrast, the 0.32 m precursor underwent seed coalescence and out‐of‐plane growth, forming well‐defined hemispherical MoS_2_ structures. The distinct morphologies of the flat MoS_2_ (*f*‐MoS_2_), intermediate MoS_2_ (*i*‐MoS_2_), and hemispherical MoS_2_ (*h*‐MoS_2_) are schematically depicted in Figure 1C.

To understand the formation mechanism of *h*‐MoS_2_, we closely examined the laser‐induced photothermal reaction of a 0.32 m precursor from the precursor film to a crystallized structure. The SEM image (Figure 1D, right), which magnifies the interfacial area between the precursor film region (yellow) and *h*‐MoS_2_ region (green) marked by the red‐dotted box in the optical microscopy (OM) image (Figure 1D, left), provides further insight into the merging behavior of the (NH_4_)_2_MoS_4_ seeds and their nucleation process. The area marked by red arrows in the SEM image indicates the initial localized region where the (NH_4_)_2_MoS_4_ seeds underwent nucleation driven by the sub‐nanosecond pulsed laser (τ ≈200 ns). Under rapid photothermal energy injection, the laser‐irradiated MoS_2_ seeds self‐heated and immediately attracted nearby precursor molecules. In the highly concentrated 0.32 m film, adjacent MoS_2_ seeds were drawn toward these regions, coalescing into thermodynamically favorable nuclei with increased nucleation density (Figure S3, Supporting Information).^[^ ^23^, ^24^ ^]^ Indeed, UV–vis spectroscopy revealed that the 0.32 m precursor had 5.72 times higher absorbance than the 0.032 m precursor at a wavelength of 1.06 µm (Figure 1E), indicating significantly enhanced light absorption under identical laser irradiation. The increased absorbance implied a higher density of thermally activated (NH_4_)_2_MoS_4_ species, which promoted localized nucleation. This resulted in the even distribution of well‐defined *h*‐MoS_2_ structures across the substrate, facilitated by the uniform irradiation of the laser beam (beam quality factor: M^2^ < 1.2), which ensured consistent photothermal energy delivery over the entire area. Furthermore, as indicated by the plot of *h*‐MoS_2_ count as a function of precursor concentration, the number of *h*‐MoS_2_ units within a 2  µm × 2 µm area increased with the precursor concentration up to 0.32 M, which was close to the maximum soluble concentration of the precursor (Figure S4, Supporting Information).

**Figure 2:**
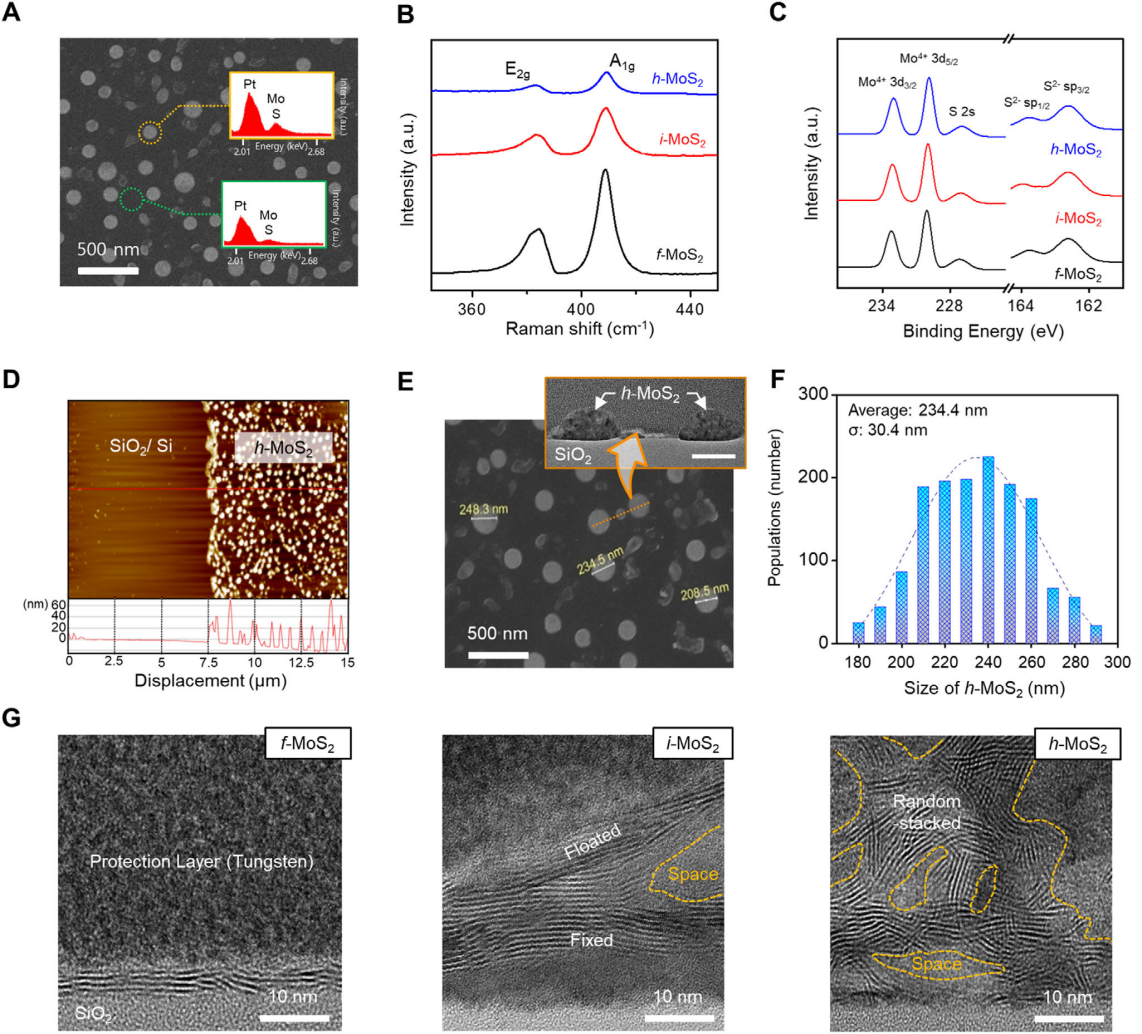
A) SEM image of laser‐synthesized *h*‐MoS_2_ and corresponding EDS spectra for Mo and S. Yellow and green dotted regions indicate bulk and adjacent planar regions of *h*‐MoS_2_, respectively. B) Raman spectra of MoS_2_ crystals synthesized from different precursor concentrations. C) XPS spectra of *f*‐MoS_2_, *i*‐MoS_2_, and *h*‐MoS_2_, showing characteristic peaks for Mo and S. D) Surface profile plot of the SiO_2_/Si substrate and hemispherical *h*‐MoS_2_ obtained from AFM. E) SEM image used to measure the diameters of *h*‐MoS_2_ structures. Inset: CS‐TEM image of *h*‐MoS_2_ (scale bar: 200 nm). F) Particle‐size distribution chart of *h*‐MoS_2_. G) Cross‐sectional HRTEM images of *f*‐MoS_2_ (left), *i*‐MoS_2_ (middle), and *h*‐MoS_2_ (right).

The chemical composition of the synthesized *h*‐MoS_2_ was investigated using energy‐dispersive X‐ray spectroscopy (EDS). Spot analysis was performed on the bulk (yellow dotted circle) and adjacent (green dotted circle) regions of *h*‐MoS_2_ (**Figure** **2**A). In both regions, overlapping Mo Lα and S Lα peaks were observed at 2.30 keV, confirming the presence of Mo and S. The bulk region exhibited a higher peak intensity, which is attributed to the local aggregation of MoS_2_ seeds during laser‐induced crystallization. This spatial variation in the Mo and S signal intensities is more clearly visualized in the EDS elemental mapping image (Figure S5, Supporting Information). Raman and X‐ray photoelectron spectroscopy (XPS) were performed to examine the chemical characteristics of the synthesized MoS_2_ crystals. Raman spectroscopy identified two characteristic vibrational modes of MoS_2_: the in‐plane E_2g_ mode and the out‐of‐plane A_1g_ mode. For *f*‐MoS_2_, *i*‐MoS_2_, and *h*‐MoS_2_, the E_2g_ and A_1g_ peaks were located at 384.47 and 408.45 cm^−1^ for *f*‐MoS_2_, 383.38 and 409.00 cm^−1^ for *i*‐MoS_2_, and 382.83 and 409.00 cm^−1^ for *h*‐MoS_2_ (Figure 2B). The increasing peak separation (|Δω| = 23.98, 25.62, and 26.17 cm^−1^) indicated a progressive increase in the number of MoS_2_ layers with higher precursor concentration.^[^ ^25^, ^26^ ^]^ This trend was supported by atomic force microscopy (AFM), which revealed that the precursor film thickness increased proportionally to the concentration: 21.3 ± 1.1 nm (0.032 M), 62.5 ± 2.2 nm (0.12 m), and 115.2 ± 5.6 nm (0.32 m) (Figure S6, Supporting Information). Thicker precursor films provided a greater supply of Mo and S species, resulting in a thicker MoS_2_ layer following laser‐induced thermolysis. Interestingly, despite the increased layer thickness, the A_1g_ mode no longer exhibited a blue shift from *i*‐MoS_2_, and the peak position remained unchanged for *h*‐MoS_2_. This spectral peak shift is attributed to the strain effects induced by the curvature and morphological changes in the MoS_2_ crystals. Such morphological transitions promote electron scattering arising from localized lattice distortions, affecting the charge‐transport characteristics. In summary, field‐effect transistor (FET) measurements revealed distinct changes in electrical behavior during the transition from *f*‐MoS_2_ to *i*‐MoS_2_. Specifically, the decrease in I_ds_ observed in both the output and transfer characteristic curves suggests a reduction in carrier mobility and the formation of potential barriers within the MoS_2_ channels, indicating the presence of lattice strain during the morphological transformation (Figure S7, Supporting Information).^[^ ^27^, ^28^, ^29^ ^]^ XPS analysis was conducted to clarify the chemical valence states of Mo and S in the synthesized MoS_2_ crystals (Figure 2C). For *h*‐MoS_2_, characteristic peaks were observed at 232.98 eV (Mo^4+^ 3d_3/2_), 229.88 eV (Mo^4+^ 3d_5/2_), 226.98 eV (S 2s), 163.78 eV (S^2−^ sp_1/2_), and 162.58 eV (S^2−^ sp_3/2_), which corresponded to the typical binding energies of 2H‐MoS_2_.^[^ ^30^, ^31^ ^]^ These peaks were also observed for *i*‐MoS_2_ and *f*‐MoS_2_, indicating that neither precursor concentration nor morphology significantly affected the chemical compositions of the MoS_2_ crystals. Furthermore, this laser‐assisted photothermal system can also be extended to synthesize hemispherical WS_2_ and Mo_x_W_1‐x_S_2_ alloys, which exhibited well‐defined chemical compositions (Figure S8, Supporting Information).

To characterize the morphological features of *h*‐MoS_2_, AFM was used to measure the particle height, which ranged from 20 to 60 nm relative to the flat surface of the SiO_2_/Si substrate (Figure 2D). The lateral dimensions were analyzed using the SEM images and ImageJ software (Figure 2E). The geometry of *h*‐MoS_2_ produced a distinct contrast and bright field in the SEM images, facilitating clear boundary identification. Cross‐sectional transmission electron microscopy (CS‐TEM) confirmed the curved structure of the crystals (inset of Figure 2E). According to this contrast, the average diameter of *h*‐MoS_2_ was determined to be 234.4 nm, with a standard deviation (σ) of 30.4 nm (Figure 2F), and the range of *h*‐MoS_2_ diameters according to the standard deviation was confirmed to include the average diameter of each of the 25 samples synthesized to assess the synthesis reproducibility (Figure S9, Supporting Information). High‐resolution transmission electron microscopy (HRTEM) was used to further investigate the structural characteristics of the synthesized MoS_2_ crystals (Figure 2G). The morphology of MoS_2_ varied significantly with the precursor concentration. For *f*‐MoS_2_ (Figure 2G, left), a continuous film‐like structure with a thickness of ≈3.5 nm was observed, corresponding to a five‐layer stacked configuration. In contrast, *i*‐MoS_2_ exhibited a partially arch‐like structure, with an internal void dividing the crystal into fixed and suspended lattice domains (Figure 2G, middle). This arch‐like morphology is attributed to the vertical tensile stress generated during laser irradiation. As the upper region of the precursor film agglomerated, out‐of‐plane stress developed perpendicular to the lateral MoS_2_ layers. This stress counteracted the van der Waals (vdW) forces between the layers, causing local delamination. In *h*‐MoS_2_ (Figure 2G, right), HRTEM revealed randomly stacked MoS_2_ layers interspersed with numerous inner voids. These features are believed to originate from the localized strain induced by Mo–S covalent bonding during hemisphere formation. A higher precursor concentration promoted the photothermal decomposition and merging of MoS_2_ seeds into a thermodynamically optimized curved structure. In this process, the Mo–S bonds adopted nonuniform orientations, diverging from the typical 2D layered arrangement. Importantly, the estimated total Mo–S covalent bonding energy (≈10^5^ J) for the 0.32 m precursor was several orders of magnitude higher than the vdW interaction energy (10^−11^–10^−10^ J) at both the SiO_2_–MoS_2_ and MoS_2_–MoS_2_ interfaces.^[^ ^32^, ^33^, ^34^ ^]^ This disparity likely introduced significant tensile stress within the MoS_2_ lattice, promoting the formation of internal voids. The types and magnitudes of the interfacial stresses are schematically shown in Figure S10 (Supporting Information).

**Figure 3:**
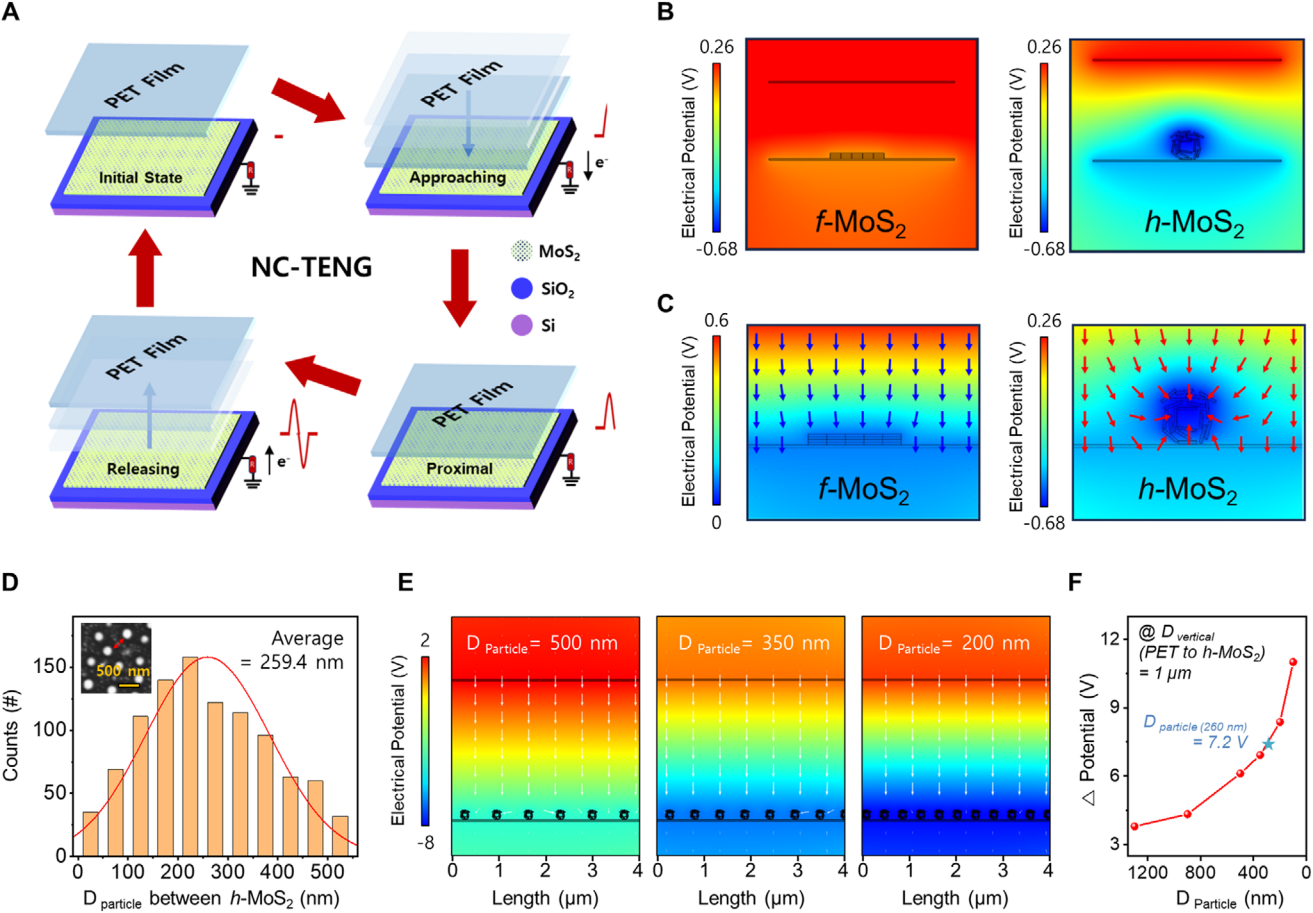
A) Schematic of the working mechanism of the *h*‐MoS_2_–based non‐contact triboelectric nanogenerator (NC‐TENG) operating in approaching–separating mode. B) COMSOL‐simulated electric‐potential distributions for single *f*‐MoS_2_ flake and single *h*‐MoS_2_ particle, rendered on a common potential scale. C) COMSOL‐simulated electric‐field magnitude and orientation for *f*‐MoS_2_ and *h*‐MoS_2_, plotted with different field‐magnitude scales to highlight localization. D) Histogram of interparticle spacing (D_Particle_) in *h*‐MoS_2_. E) COMSOL‐simulated electric‐potential distributions for *h*‐MoS_2_ with varied interparticle spacing. F) Calculated electric‐potential difference as a function of *h*‐MoS_2_ interparticle spacing.

Leveraging its unique curved morphology and intrinsic negative triboelectric polarity, *h*‐MoS_2_ was employed as the triboelectric negative layer in a NC‐TENG device. PET was selected as the counter layer because of its strong tendency to acquire positive charges upon interaction.^[^ ^35^ ^]^ As illustrated in **Figure** **3**A, the device operates in a non‐contact mode, where electrical signals are generated via electrostatic induction, and the required initial charges were established by contacting PET film with the *h*‐MoS_2_ surface using a digital indicator.^[^ ^36^ ^]^ In the initial state, both PET and *h*‐MoS_2_ remain stationary, and no charge transfer occurs. However, when the PET film approaches the electrified *h*‐MoS_2_ surface, electrostatic induction leads to the accumulation of negative charges on the *h*‐MoS_2_ surface, resulting in a potential difference. This potential difference drives electrons to flow through an external circuit connected to the ground and back, completing a full triboelectric cycle without direct physical contact between the two layers.^[^ ^37^ ^]^

To evaluate how the morphology of MoS_2_ crystals affects the performance of the NC‐TENG, the electric‐field distribution was simulated using the finite‐element method (FEM) in COMSOL Multiphysics. The simulation environment replicated the operational conditions of an actual device by incorporating a PET film, SiO_2_ substrate, and MoS_2_ crystal layers. This setup allowed a comparative analysis of the electric‐field concentration across different MoS_2_ morphologies. The detailed parameters and boundary conditions used in the simulation are presented in Section 4. The structural advantage of *h*‐MoS_2_ crystals in modulating the induced electric field was evident in the finite‐element simulations (Figure 3B). At an identical separation distance (d ≈ 500 nm) between the MoS_2_ surface and the PET film, the potential distribution map revealed a substantially higher electrical potential for *h*‐MoS_2_ than for *f*‐MoS_2_. Although the *f*‐MoS_2_ system exhibited minimal contrast with the PET layer, resulting in a uniform red hue across the domain, the *h*‐MoS_2_ system displayed a distinct color gradient, indicating a sharper potential difference. Specifically, the maximum potential difference reached 0.94 V for *h*‐MoS_2_, whereas it was only 0.16 V for *f*‐MoS_2_ (Figure S11, Supporting Information). The electric‐field distribution confirmed this trend (Figure 3C). In the case of *f*‐MoS_2_, the electric field was broad and weakly concentrated because of the flat morphology and absence of internal voids (Figure 3C, left; blue arrows). In contrast, *h*‐MoS_2_ produced a highly localized and intensified electric field (Figure 3C, right; red arrows). This enhancement is attributed to its curved hemispherical geometry and internal voids, which collectively increase the surface curvature and electron‐capturing efficiency.^[^ ^38^, ^39^ ^]^ Building on this baseline comparison, we expanded the FEM simulations from a single‐particle model to multi‐particle assemblies to more realistically capture the morphology of *h*‐MoS_2_. SEM analysis (inset of Figure 3D) revealed that the laser‐irradiated regions are composed of hemispherical particle assemblies with a few‐hundred nanometer spacing (≈260 nm on average). To capture this structural feature, we explicitly introduced the interparticle spacing (D_particle_) as a simulation parameter. As shown in Figure 3E, reducing D_particle_ from 500 to 200 nm led to a marked increase in the maximum potential difference between *h*‐MoS_2_ and PET. The systematic results across a wide range of interparticle spacings (1300 nm to 100 nm), summarized in Figure 3F, further demonstrate that at a fixed vertical separation of 1 µm, the potential difference rises from ≈0.94 V in the single‐particle model to ≈7.2 V at the experimentally relevant spacing of ≈260 nm (≈7.7‐fold), and further to ≈9.8 V at the smallest spacing considered. This enhancement originates from constructive field coupling between adjacent particles, whereby initially circular potential profiles overlap and evolve into plate‐like distributions, reinforcing the overall electrostatic field.

Taken together, these simulation results demonstrate that the unique morphology of *h*‐MoS_2_, arising not only from its hemispherical curvature and internal cavities but also from the closely spaced particle assemblies and interparticle coupling—significantly amplifies the electrostatic induction potential during noncontact operation, consistent with previous experimental studies reporting morphology‐driven enhancement of triboelectric performance.^[^ ^40^, ^41^ ^]^

**Figure 4:**
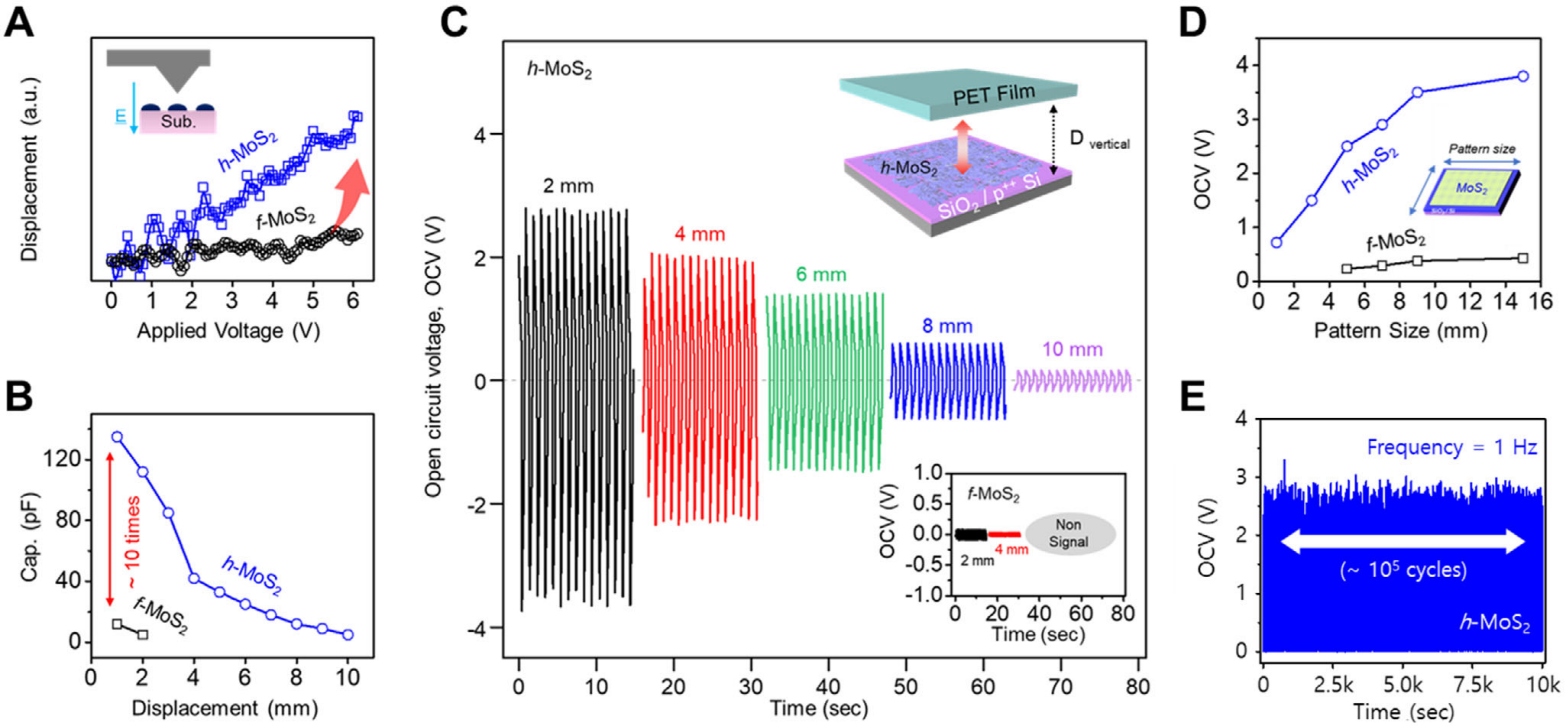
A) PFM analysis of *f*‐MoS_2_ and *h*‐MoS_2_, showing differences in electromechanical coupling behavior. B) Capacitance measurements of *f*‐MoS_2_ and *h*‐MoS_2_ as a function of displacement from the PET film. C) OCV of the *h*‐MoS_2_‐based NC‐TENG measured under a fixed actuation frequency of 1 Hz and varying vertical distances (D_vertical_). Inset: OCV of the *f*‐MoS_2_‐based NC‐TENG under the same conditions. D) Output voltages of *f*‐MoS_2_ and *h*‐MoS_2_ devices as a function of the laser‐patterned active area (D_vertical_ = 2 mm). E) 10,000 cycles durability test of the *h*‐MoS_2_‐based NC‐TENG under fixed operation conditions (frequency: 1 Hz, D_vertical_: 1 mm).

The electromechanical behaviors of *f*‐MoS_2_ and *h*‐MoS_2_ were characterized using piezoresponse force microscopy (PFM) (**Figure** **4**A). Although multilayer MoS_2_ lacks intrinsic piezoelectricity, out‐of‐plane PFM displacement signals can arise owing to electrostatic charging effects.^[^ ^42^ ^]^ Notably, the slope of the piezoresponse–voltage curve, which corresponds to the effective piezoelectric coefficient (d_33_), was 6.07 times higher for *h*‐MoS_2_ than for *f*‐MoS_2_. This remarkable enhancement is attributed to localized charge accumulation within interfacial voids and the concentrated electric field induced by the curved morphology of *h*‐MoS_2_. These factors intensify internal electric fields and promote polarization under an applied bias, amplifying the PFM response.^[^ ^20^, ^36^ ^]^ These findings are in direct agreement with the FEM results in Figure 3C–E, where hemispherical particle geometries were shown to intensify local electrostatic potentials, thereby amplifying the electromechanical response.

To assess the noncontact operating range and the corresponding electrical response, we measured the capacitances of *f*‐MoS_2_ and *h*‐MoS_2_ using an LCR meter while gradually increasing the distance between the PET film and the MoS_2_ crystals (Figure 4B). For *f*‐MoS_2_, the capacitance dropped from 12 pF at 1 mm to 5 pF at 2 mm and was undetectable beyond 3 mm. In contrast, *h*‐MoS_2_ exhibited significantly higher capacitance values of 135 pF (1 mm) and 112 pF (2 mm), maintaining measurable signals up to 10 mm. This long‐range capacitive response in *h*‐MoS_2_ is consistent with the FEM results in Figure 3F, which demonstrated that constructive field coupling in multi‐particle assemblies extends the effective electrostatic induction. Considering this result, the OCV was measured by vertically oscillating the PET film (1 Hz) over increasing distances (Figure 4C). The *f*‐MoS_2_‐based NC‐TENG generated OCVs of 0.29 V (2 mm) and 0.06 V (4 mm), with no measurable output beyond 4 mm. In contrast, *h*‐MoS_2_‐based devices exhibited OCVs of 2.01 V (2 mm), 1.48 V (4 mm), and 0.25 V even at 10 mm, demonstrating a greatly extended and stable operating range. We also evaluated the device performance as a function of the active‐area pattern size (Figure 4D). In the case of *f*‐MoS_2_, the OCV increased gradually from 0.23 V (5 mm) to 0.43 V (15 mm) with increasing active area. In comparison, *h*‐MoS_2_ exhibited a far steeper rise in OCV, increasing from 0.72 V (1 mm) to 3.78 V (15 mm), which indicated a significantly higher sensitivity to charges in the active area. This pronounced response was likely due to enhanced electric‐field localization in the hemispherical MoS_2_ structure. Finally, a durability test was conducted on the *h*‐MoS_2_‐based NC‐TENG at D_vertical_ = 1 mm and 1 Hz (Figure 4E). Owing to its contact‐free operation, the device maintained a stable voltage output during 10,000 cycles, confirming its excellent operational stability under the typical humidity conditions (RH≈40%). In addition, extended stability tests demonstrated that the hemispherical structure preserved stable triboelectric performance even under humid conditions (RH ≈70%). Moreover, preliminary measurements indicated that comparable stability was achieved when integrating WS_2_ and Mo_x_W_1−x_S_2_ alloys with different polymeric layers (Figure S12, Supporting Information).

**Figure 5:**
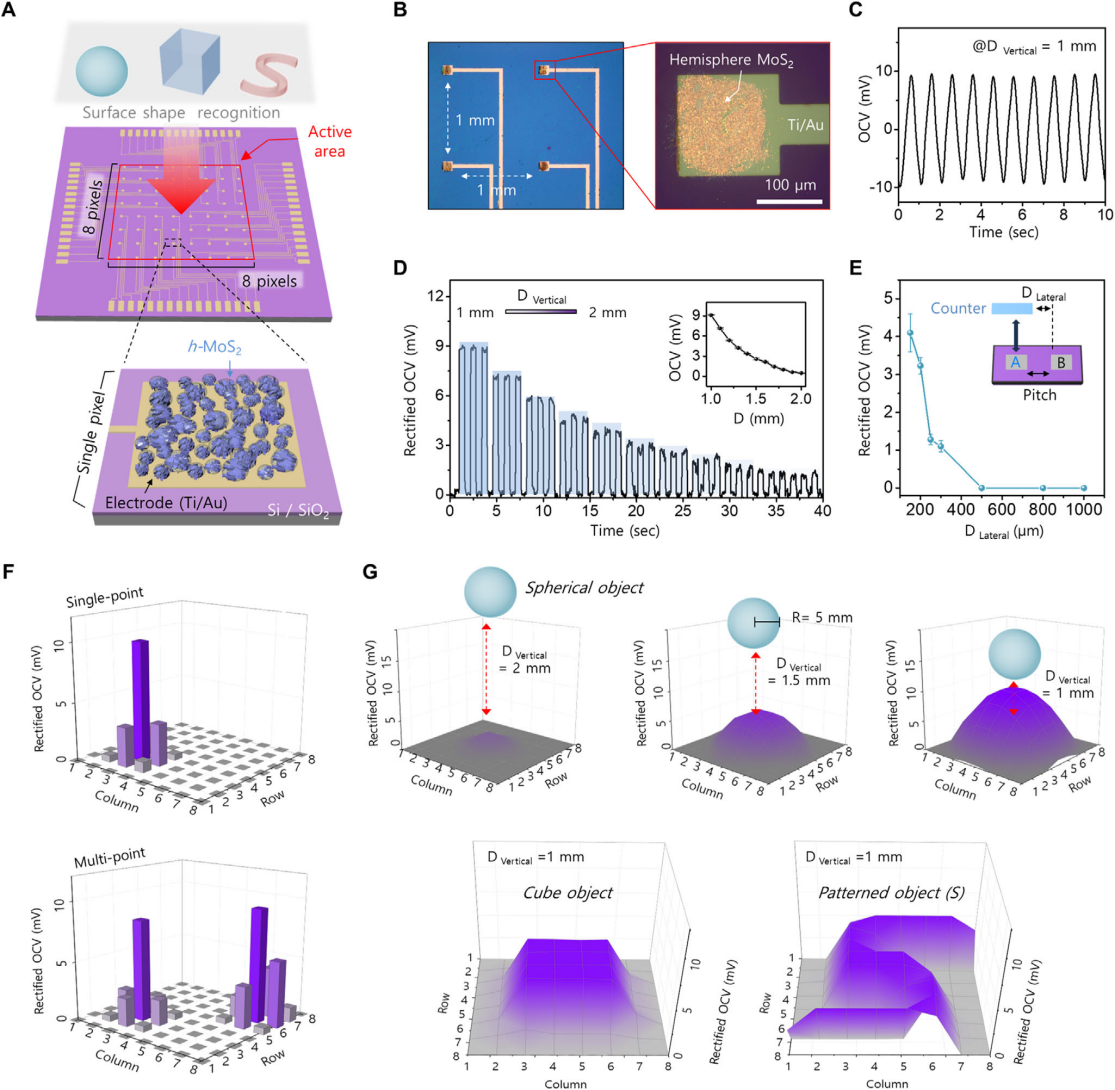
A) Schematic of the noncontact sensing system comprising an 8 × 8 *h*‐MoS_2_ array for remote object recognition. Bottom inset: magnified view of a single pixel structure. B) OM image of the *h*‐MoS_2_ integrated metal electrode circuit. Pixel dimensions are shown in the magnified view. C) OCV response generated from a single pixel. D) Rectified OCV signal of a single pixel with varying vertical distances over time. Inset: Corresponding OCV response to time‐dependent distance changes. E) Summary of pixel‐pitch‐dependent crosstalk in the *h*‐MoS_2_ array. F) Coordinate‐based sensing of single‐ and dual‐point vertical stimuli, visualized as 3D rectified OCV bar plots at defined pixel locations. G) Pattern‐recognition results for various 3D‐printed counter objects, including geometric (sphere, cube) and alphabetic (S‐shape) forms, captured by the noncontact sensor array.

This laser‐directed strategy enabled the fabrication of a miniaturized and contactless *h*‐MoS_2_ sensor array. As a demonstration, an 8 × 8 *h*‐MoS_2_ pixel array was integrated onto a patterned Au/Ti electrode over a 1 × 1 cm^2^ active area (**Figure** **5**A). The patterned metal electrode was fabricated via photolithography, and the *h*‐MoS_2_ film was transferred using a pressure‐sensitive adhesive film (PSAF) (Figure S13, Supporting Information). Given that the laser‐directed strategy allows the synthesis of *h*‐MoS_2_ with a minimum size of 100 µm × 100 µm (Figure S14, Supporting Information), each pixel was designed with dimensions of 120 µm × 120 µm and served as a unit cell of a miniaturized image sensor (Figure 5B). To evaluate the sensing capability of individual pixels, a PET counter material was sinusoidally approached and retracted at 1 Hz with a vertical amplitude (D_vertical_) of 1 mm. A stable OCV signal of ≈10 mV was generated during this process, with the reduced amplitude attributed to the limited active area contributing to surface electrification (Figure 5C). A distance‐dependent trend was also confirmed, with the OCV signal decreasing gradually as the vertical distance increased from 1 to 2 mm in 0.1‐mm increments (Figure 5D). These results demonstrate that the pixelated *h*‐MoS_2_ retains effective surface charge induction, even at the microscale It should be noted that Figure 5C shows the intrinsic alternating OCV signal from a single pixel, whereas Figure 5D–F present the unipolar envelopes obtained after sequential signal conditioning using an OP‐amp‐based rectifier and an MCU‐controlled multiplexer chain, enabling reliable array‐level readout. The overall circuit flow and component configuration are summarized in Figure S15 (Supporting Information). To further validate the sensing capability for a non‐contact image sensor, we additionally observed the signal crosstalk behavior of the *h*‐MoS_2_ array with various pixel pitch distances (D _Lateral_). The fabricated device image of the *h*‐MoS_2_ array with 100, 300, 500, and 1000 µm pitch, and the observed pixel crosstalk results, are shown in Figure S16 (Supporting Information). As summarized in Figure 5E, a selective response without crosstalk could be achieved when the two adjacent pixels (A and B) were separated above 500 µm. This confirmed the spatial resolution and selectivity of the sensor array. Furthermore, leveraging this optimized pitch condition, we evaluated the sensing capability toward micro‐scale stimuli by vertically actuating a polymer‐coated micro‐tip (Ø = 450 µm) at four different pixel coordinates (Figure S17, Supporting Information). Distinct and crosstalk‐free OCV responses were observed only at the stimulated pixels, thereby validating that the array can reliably detect objects with diameters of several hundred micrometers without interference. These findings highlight the scalability and precision of the proposed *h*‐MoS_2_‐based sensing platform and pave the way for its application in next‐generation contactless sensing systems with high‐density array configurations.

As a proof of concept, we evaluated the contactless sensing capabilities of the *h*‐MoS_2_‐based array under various contactless stimuli and bulk object geometries (≈ mm scale) (Figure S18, Supporting Information). To assess the multi‐pixel response behavior, a 3D‐printed rounded tip (Ø = 3 mm) was vertically actuated toward the array at a D_vertical_ of 1 mm. As indicated by the single‐point OCV response in Figure 5F, distinct OCV signals were detected at the pixels directly beneath the contact point, whereas weaker responses from neighboring pixels were attributed to slight height differences caused by the curved surface of the tip. Considering the optimized D_Lateral_ geometry of the *h*‐MoS_2_ array (pixel pitch > 500 µm), these weak responses from neighboring pixels are not the result of crosstalk but rather originate from the geometric curvature of the counter object. Under dual‐point stimuli, the array produced spatially separated responses, demonstrating its ability to simultaneously resolve multiple inputs. This pixel‐level OCV variation enabled not only event localization but also the differentiation of stimuli based on object geometry and distance. Figure 5G presents the real‐time position‐mapping results with various 3D‐printed objects placed at the center of the array. For a spherical object (radius: 5 mm), spatially graded OCV outputs were observed across the array, reflecting curvature‐dependent changes in vertical distance. In contrast, a cubic object (5 × 5 mm^2^) generated square‐shaped signal patterns with uniform intensity, clearly revealing its flat geometry. Notably, the system successfully recognized more complex patterns. When an S‐shaped object approached the array, pixel‐wise OCV mapping accurately reproduced the curved profile of the letter. These demonstrations underscore the potential of laser‐synthesized, high‐capacitance 2D materials for shape‐resolved, contactless sensing, a domain traditionally dominated by optoelectronics and difficult to implement without physical contact or optical components.

## 3 Conclusion

We achieved the one‐step laser‐assisted synthesis of hemispherical MoS_2_ (*h*‐MoS_2_) nanostructures and demonstrated their effectiveness as high‐performance, noncontact triboelectric layers. Compared with conventional flat MoS_2_, hemispherical MoS_2_ exhibited an increased electric‐field concentration owing to its curved morphology and internal voids, leading to substantial improvements in capacitance, OCV, and operating distance in NC‐TENG devices. Finite‐element simulations and experimental measurements revealed that the unique structural features of *h*‐MoS_2_ significantly amplified the electrostatic induction. The *h*‐MoS_2_‐based NC‐TENG achieved a 22‐fold increase in capacitance and a 37‐fold increase in OCV compared with its flat counterpart while operating stably at distances of up to 10 mm. Furthermore, a pixelated *h*‐MoS_2_ sensor array fabricated via laser‐directed synthesis demonstrated reliable contactless sensing with high spatial resolution and a shape‐recognition capability. These findings highlight the potential of scalable geometry‐engineered 2D materials for next‐generation energy‐harvesting and intelligent sensing platforms, beyond the limitations of conventional 2D architectures and optoelectronic systems. The demonstrated integration of laser patterning, structural control, and system‐level applications paves the way for advanced noncontact sensor architectures and flexible electronics.

## 4 Experimental Section

### Precursor Preparation and Laser‐assisted Synthesis

A highly doped SiO_2_/Si wafer (300‐nm‐thick SiO_2_ dielectric layer) was sonicated in ethanol and deionized water for 10 min. O_2_ plasma treatment was conducted at 100 W for 100 s to enhance the hydrophilicity of the substrate. To prepare aqueous‐type precursors with various concentrations (0.032 m, 0.12 m, and 0.32 m), (NH_4_)_2_MoS_4_ was dissolved in the mixed organic solution (dimethylformamide (DMF):n‐butylamine:2‐aminoethanol = 5:2:1, volume ratio), followed by sonication at 50 °C for 30 min. After synthesis of the precursor, the prepared solution was drop‐casted onto the O_2_ plasma‐treated SiO_2_/Si wafer and spin‐coated at 500 and 2500 rpm for 10 and 30 s, respectively. Then, the precursor film was heated 150 °C for 3 min. Subsequently, the precursor film was thermolyzed using the photothermal energy of a pulsed fiber laser (Universal Laser Systems, pulse duration of ≈200 ns with 50‐kHz repetition rate). Finally, the specimen was immersed in DMF at 130 °C for 10 min to remove the untreated precursor residue.

### Morphology‐Dependent Electric‐Field Simulation of MoS_2_ Structure

The electrostatic characteristics of *f*‐MoS_2_ and *h*‐MoS_2_ were investigated through FEM analysis using COMSOL Multiphysics software. The simulation was conducted in 2D using an electrostatic module. *f*‐MoS_2_ was modeled as a flat, stacked multilayer structure, and *h*‐MoS_2_ was represented as an aggregated multilayer structure containing internal voids. Each model consisted of ≈20 mesh elements (*f*‐MoS_2_:20 meshes; *h*‐MoS_2_:19 meshes), with each element having a lateral width of 70 nm and a vertical height of 10 nm. To simulate electrostatic induction and the formation of the electric‐field cloud, dielectric constants were assigned to the configuration elements: 15.01 for MoS_2_ and 3.50 for the PET film.^[^ ^43^, ^44^, ^45^ ^]^ The potential difference and electric‐field distribution were evaluated by controlling the distance between the PET film and the fixed MoS_2_ crystal surface from 10 to 500 nm, as visualized through a colored scale bar.

### 3D Printing of Polymeric Objects and Measurements of Noncontact Sensing Signals

The detecting objects, modeled using Autodesk Fusion, were 3D‐printed using a triboelectrically positive polylactic acid filament (CooBeen, 1.75 mm ± 0.2 mm, 3 kg) on a Creality M1 Max 3D printer (∅ = 0.4 mm).^[^ ^46^ ^]^ The slice thickness was set to 0.2 mm, with a nozzle temperature of 235 °C and a bed temperature of 50 °C. The printing speed was adjusted according to the object geometry: S‐pattern and box‐shaped objects were printed at 200 mm s^−1^, rounded tips were printed at 82–200 mm s^−1^, and spherical objects were printed at 10–200 mm s^−1^. The approaching and receding movements of the counterpart materials (PET film and 3D‐printed objects) were controlled using a digital indicator (Bongshin, BS‐3520), while the position and vertical distance between the object and the sensing system were adjusted using a customized program. The raw OCV signals from a single *h*‐MoS_2_ TENG were monitored in real time using a nanovoltmeter (Keithley 2182A). Signals from the *h*‐MoS_2_ pixels were sequentially acquired via four digital multiplexers (16‐to‐1). The raw OCV signals were then differentially amplified at a gain (×50) using an instrumentation amplifier (AD620), followed by precision rectification (AD8606). The conditioned outputs were digitized using an MCU (Arduino Due) and transferred to the PC for LabVIEW‐based visualization and object‐pattern recognition, where baseline correction and normalization were performed to ensure consistent pixel‐to‐pixel comparison.

## Conflict of Interest

The authors declare no conflict of interest.

## Supporting information



Supporting Information

## Data Availability

The data that support the findings of this study are available in the supplementary material of this article.
