# Supplementary material for: Laser‐Induced Photothermal Conversion to Hemispherical MoS2 Enabling Non‐Contact Self‐Powering Image Sensor
Source: Adv Sci (Weinh). 2025 Sep 8;12(47):e13887. doi: 10.1002/advs.202513887 (PMC12713067; doi:10.1002/advs.202513887)
Supplement: Supplementary file 1 — Supporting Information [file ADVS-12-e13887-s001.docx]

Supporting Information

Laser-Induced Photothermal Conversion to Hemispherical MoS_2_ Enabling Non-Contact Self-Powering Image Sensor

Chan-Jin Kim^†^, Kwang-Hun Choi^†^, Taegyeong Lim^†^, Seokjun Cha, Yoon-ho Lee, Da Som Song, Ye Jun Lee, Sunhwa Hong, Jun Ho Kim, Seung Hoon Lee, Saewon Kang, Wooseok Song, Sung Myung, Jongsun Lim, Sun Sook Lee, Ki-Seok Ahn, Soonmin Yim^*^, Byung Hee Hong^*^, and Sungwoong Park^*^


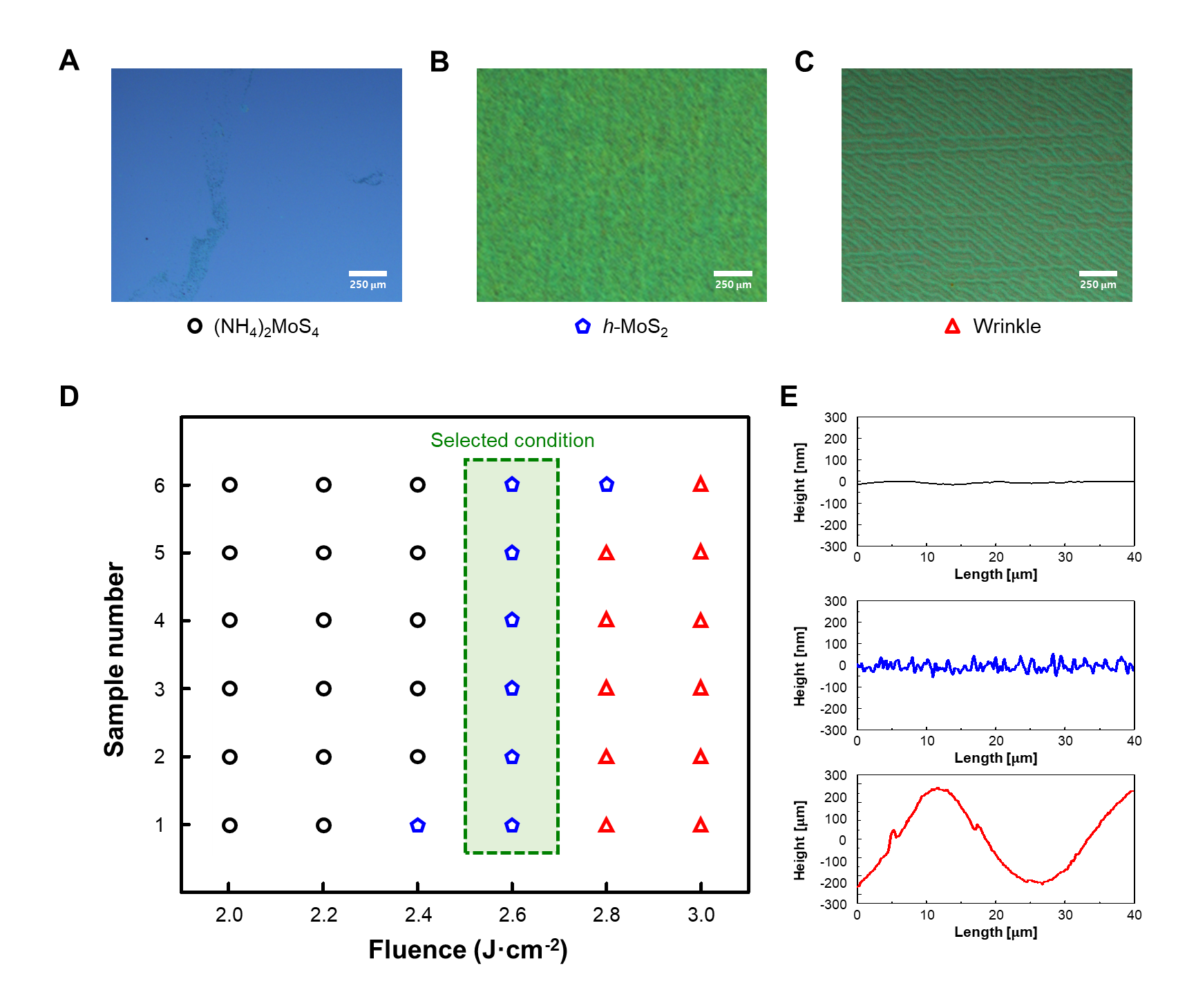


**Figure S1.** OM image of three cases of laser-irradiated precursor film showing A) SiO_2_/Si wafer after washing with DMF, B) successfully thermolyzed into *h*-MoS_2_, C) wrinkled *h*-MoS_2_ with the deformation of the SiO_2_/Si substrate by excessive photothermal energy. D) Phase diagram of the synthesized MoS_2_ structure according to the level of laser fluence. E) Surficial profile images of undecomposed (NH_4_)_2_MoS_4_ washed-out SiO_2_/Si surface by DMF (top), successfully synthesized *h*-MoS_2_ (middle), and synthesized *h*-MoS_2_ on the wrinkled substrate (bottom), obtained from AFM analysis.

In this study, the obtained results after photo-irradiation process could be categorized into three cases according to the laser fluence level: (1) incomplete thermolysis of (NH_4_)_2_MoS_4_ due to the insufficient photothermal energy, resulting in removal of precursor film after DMF rinsing process (Figure S1A), (2) uniform and continuous photothermal conversion to MoS_2_ (Figure S1B), (3) formation of the wrinkled structure due to the deformation of SiO_2_/Si wafer caused by excessive thermal energy (Figure S1C). As shown in the phase diagram (Figure S1D), MoS_2_ was not synthesized under the fluence levels below 2.4 J/cm^2^, and micrometer-scale wrinkled structure was formed at the fluence levels above 2.8 J/cm^2^ (Figure S1E). Only at the level of 2.6 J/cm^2^, the precursor film was continuously thermolyzed into MoS_2_ without any substrate deformation only at the level of 2.6 J/cm^2^, and the laser fluence was fixed to 2.6 J/cm^2^.

**
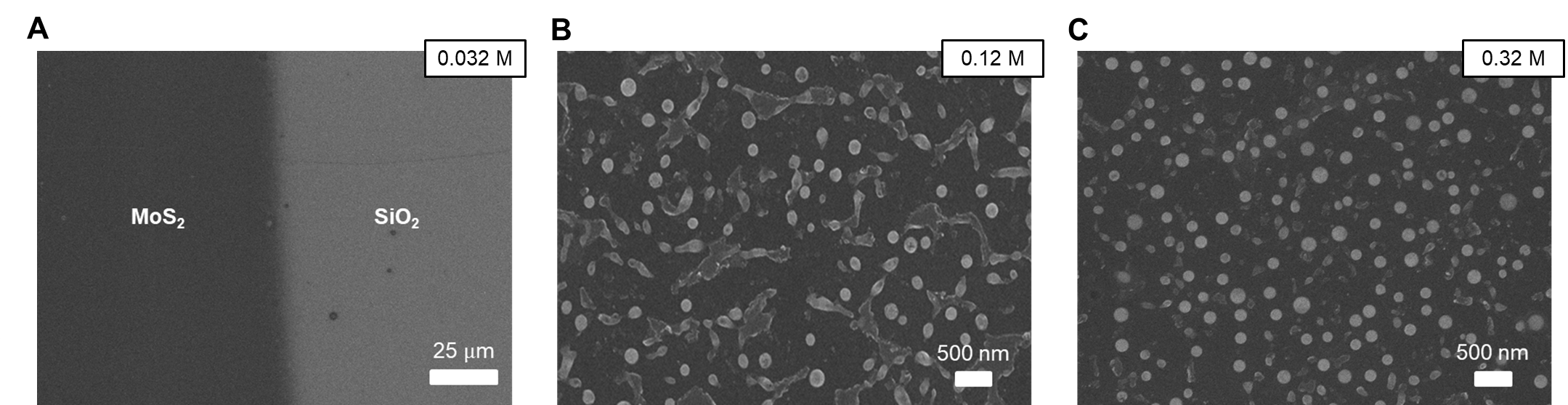
**

**Figure S2.** SEM images of the thermolyzed MoS_2_ surfaces from A) 0.032 M, B) 0.12 M, and C) 0.32 M MoS_2_ precursors.


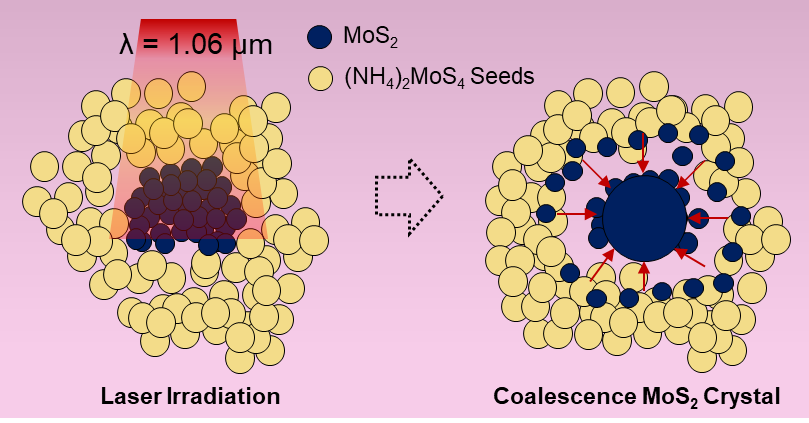


**Figure S3.** Schematic of the synthesis mechanism of *h*-MoS_2_ based on the instantaneous photothermal energy.


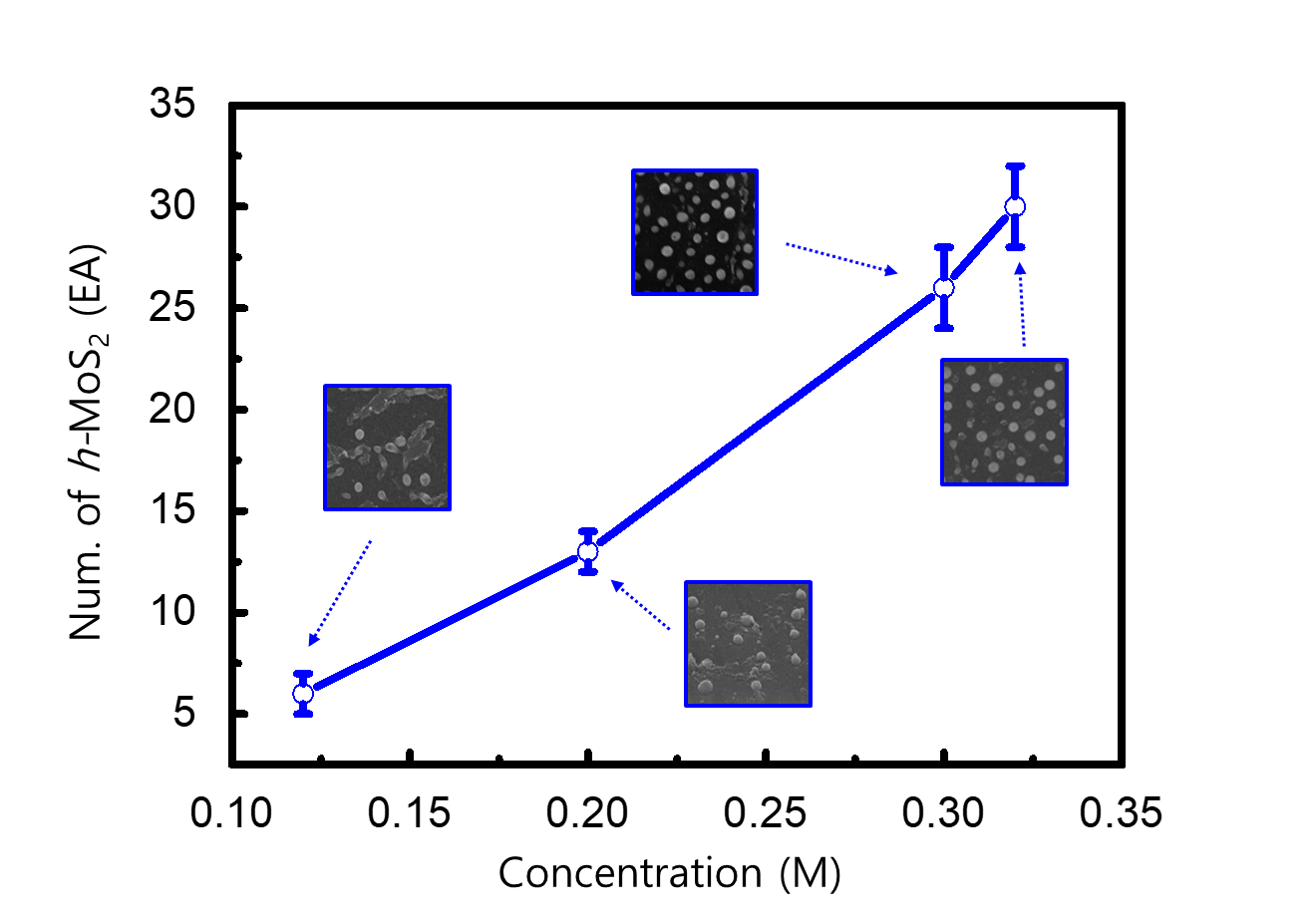


**Figure S4.** Number of *h*-MoS_2_ per 2 $\mu$m x 2 $\mu$m area as a function of precursor concentration (Inset image: representative SEM images of *h*-MoS_2_ synthesized from each precursor concentration).


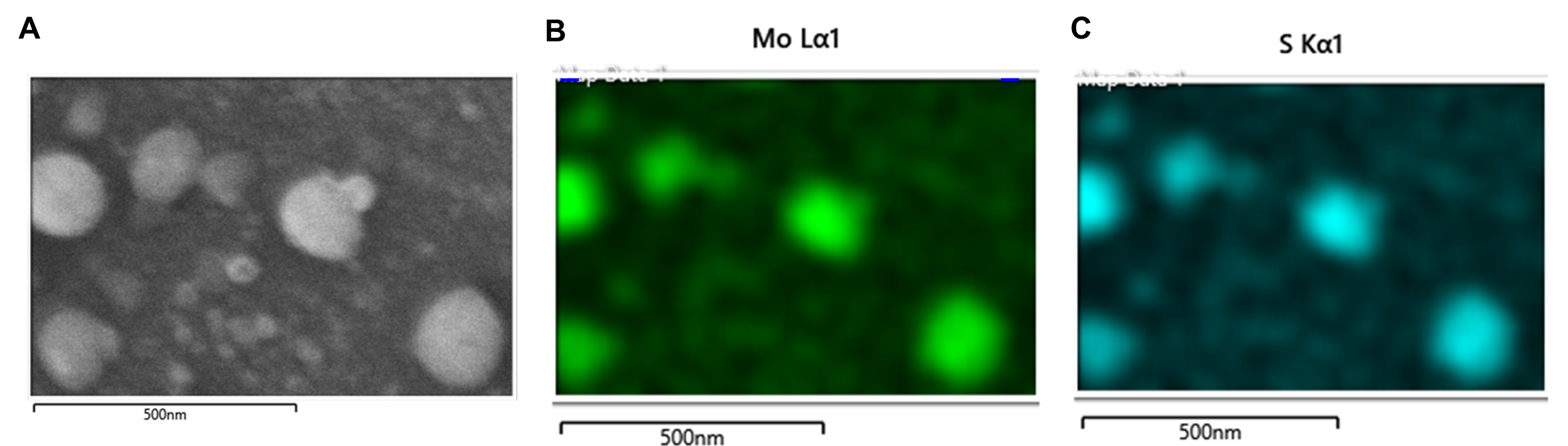


**Figure S5.** A) SEM image and EDS mapping images of *h*-MoS_2_ on the SiO_2_ wafer showing the elemental distribution of B) Mo and C) S.


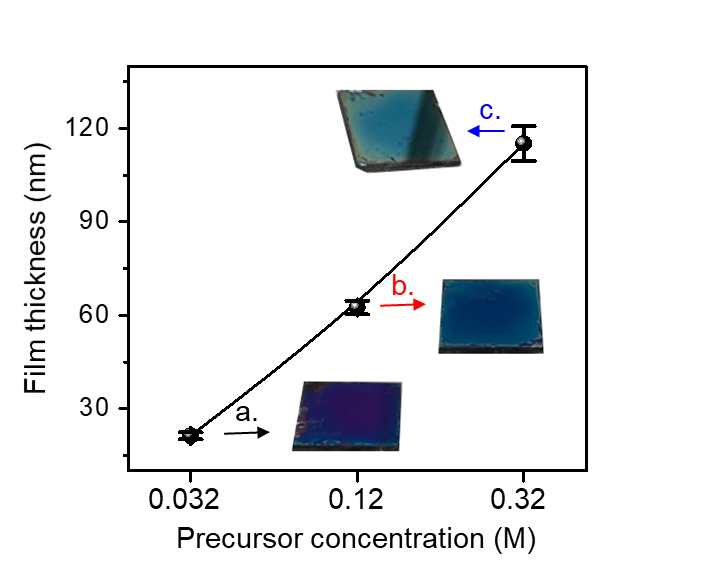


**Figure S6.** Thickness of the spin-coated precursor film for the different precursor concentrations (a: 0.032 M, b: 0.12 M, c: 0.32 M).


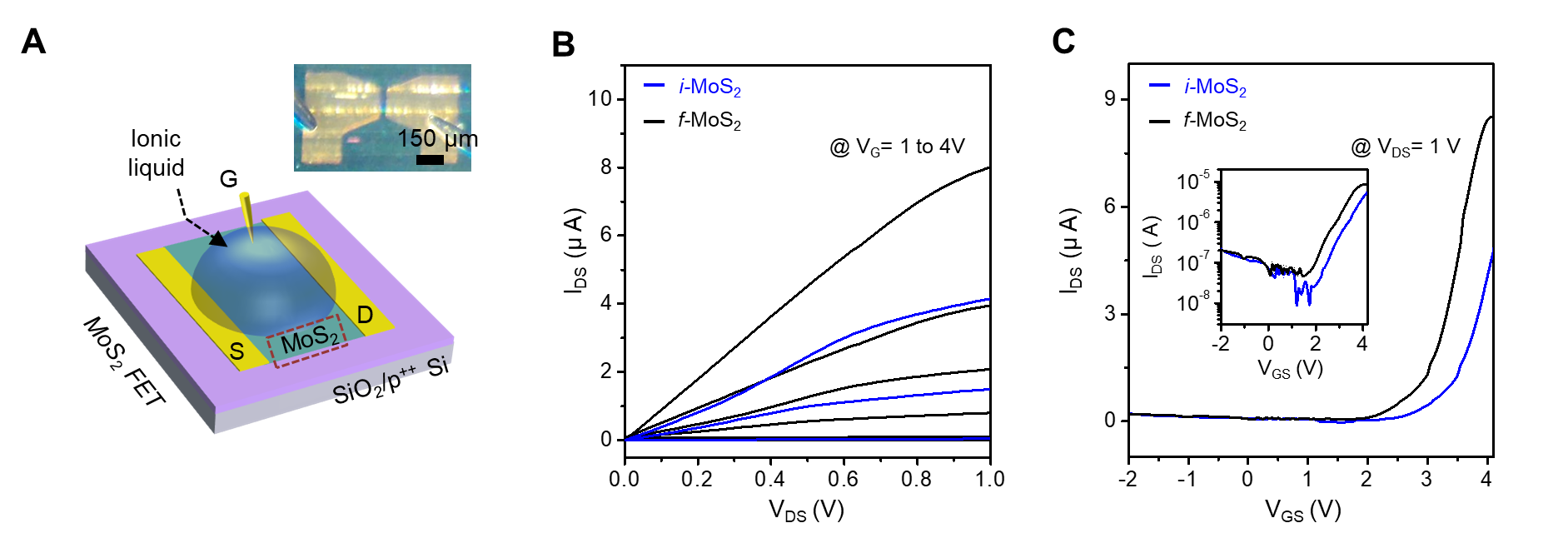


**Figure S7.** A) Schematic of the fabricated FET and the electrical-property measurement method. B) Output characteristic curves (V_DS_ vs. I_DS_) of *f*-MoS_2_ and *i*-MoS_2_ with different gate voltages: 1, 2, 3, and 4 V. C) Transfer curves (I­_DS_ vs. V_G_) of *f*-MoS_2_ and *i*-MoS_2_ measured at V_DS_ = 1 V on a linear scale (inset: corresponding transfer curves on a logarithmic scale).

To further investigate the electrical characteristics of the synthesized MoS_2_ crystals, FET measurements were performed on devices fabricated with *f*-MoS_2_ and *i*-MoS_2_. Both samples exhibited typical n-type behaviors; however, noticeable differences were observed in their transfer characteristics. Compared with *f*-MoS_2_, the *i*-MoS_2_ device exhibited a broader transfer curve and reduced on/off current ratio, suggesting degraded gate modulation. This change is attributed to the presence of tensile strain in the *i*-MoS_2_ lattice induced during the morphological transition from flat to partially curved structures. Interestingly, despite the reduced switching performance, the field-effect mobility of *i*-MoS_2_ was moderately enhanced, likely owing to the strain-induced suppression of intervalley scattering and improved carrier delocalization. These observations support the presence of structural strain in *i*-MoS_2_ and correlate well with the blue shift observed *via* Raman spectroscopy, reinforcing the influence of morphology-induced lattice deformation on the electronic properties of MoS_2_.


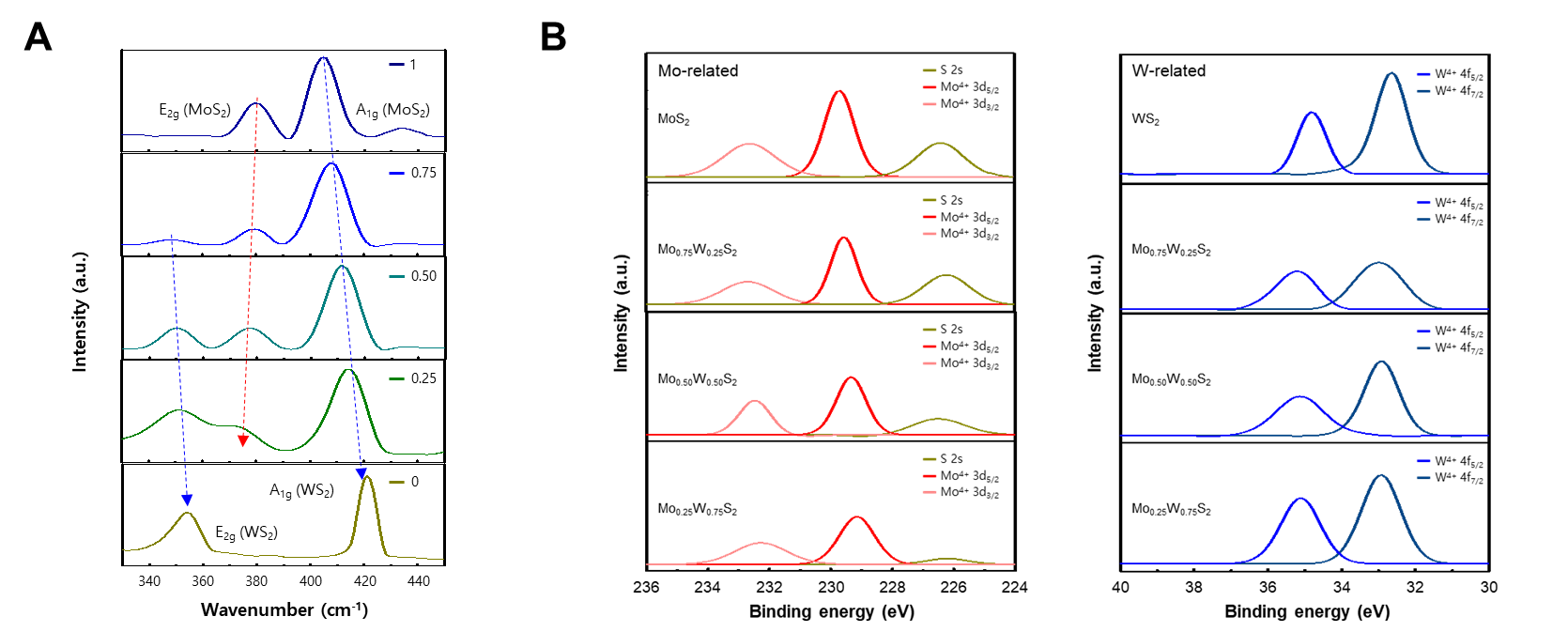


**Figure S8.** A) Raman spectra of Mo_x_W_1-x_S_2_ (x = 0~1) alloys showing three dominant peaks: E_2g_ (MoS_2_), E_2g_ (WS_2_), and the combined A_1g_ (MoS_2_ + WS_2_). B) XPS spectra of Mo_x_W_1-x_S_2_ (x = 0~1) indicating the Mo-related (left) and W-related (right) orbital states.


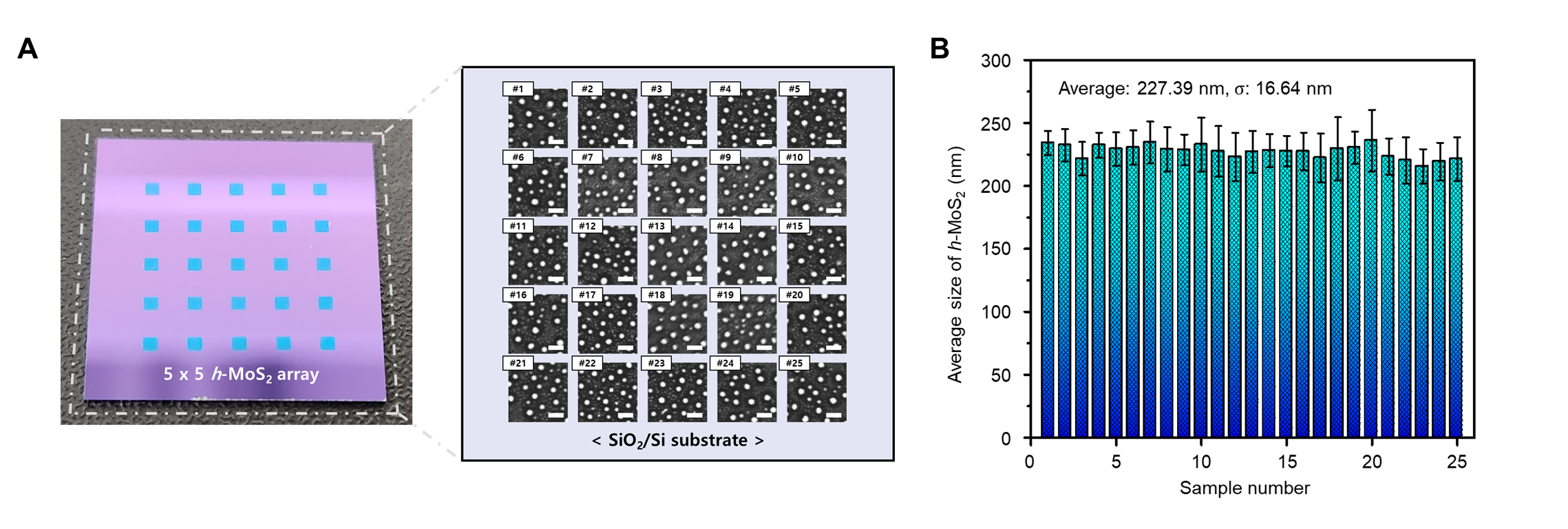


**Figure S9.** A) Photograph of 5 x 5 array of 1 mm x 1mm *h*-MoS_2_ pixels synthesized on a 2 cm x 2 cm SiO_2_/Si substrate (left), and representative SEM images for each of 5 x 5 array *h*-MoS_2_ samples (right, scale bar: 500 nm). B) Plot of the average size of *h*-MoS_2_ for each sample number with the corresponding standard deviation range.


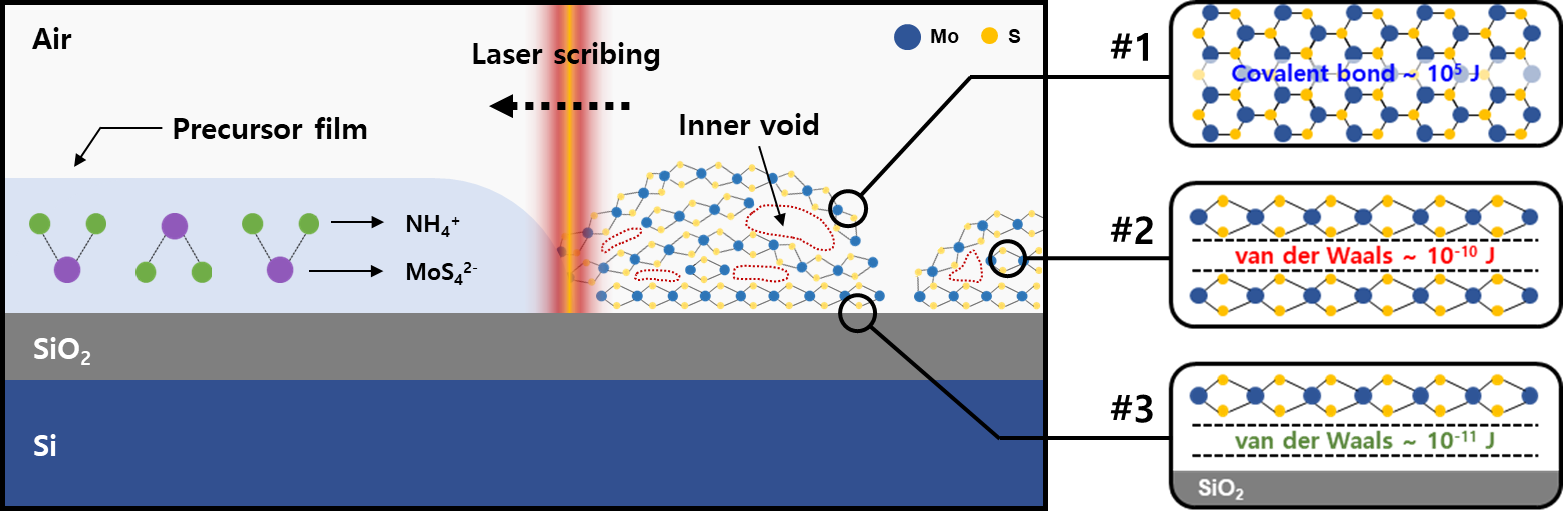


**Figure S10.** Schematic of the interaction energy scale at each interface of *h*-MoS_2_.


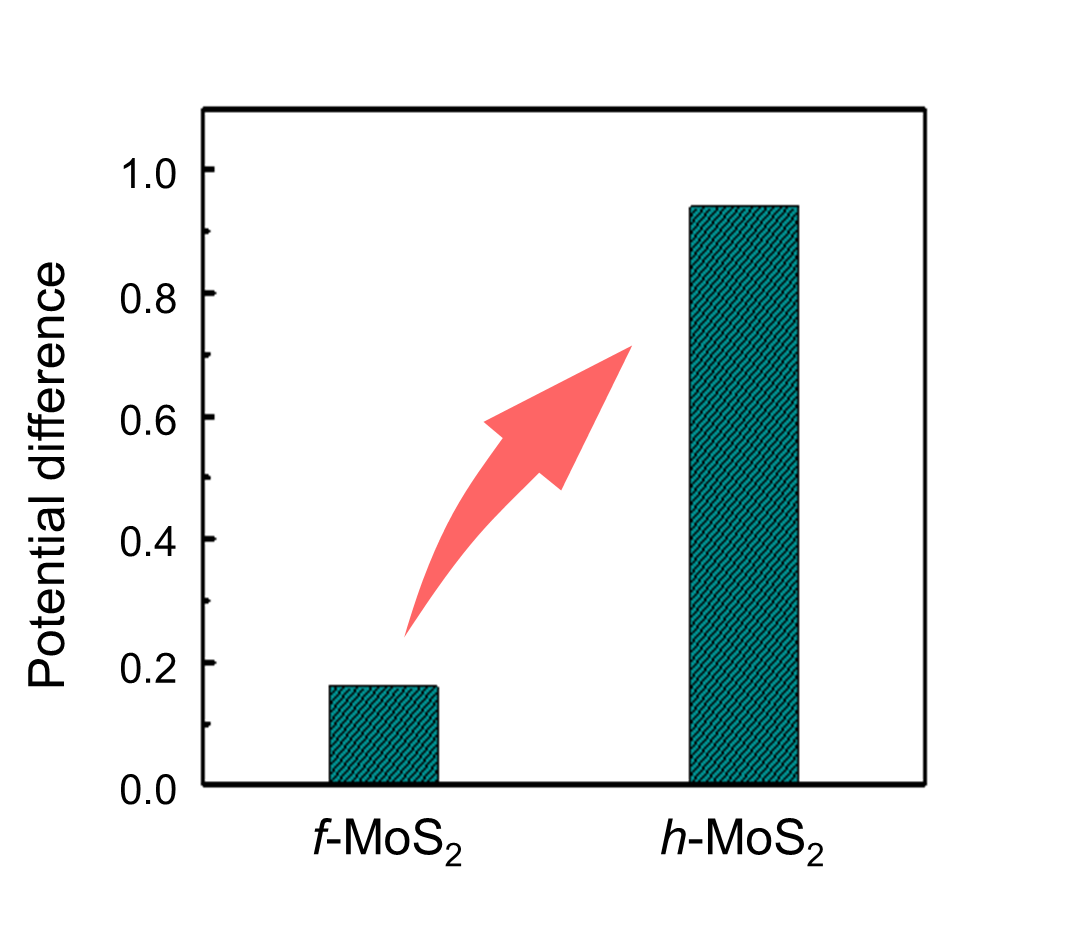


**Figure S11.** Comparison chart of the potential difference of *f*-MoS_2_ and *h*-MoS_2_ measured in the COMSOL electric-field simulation.


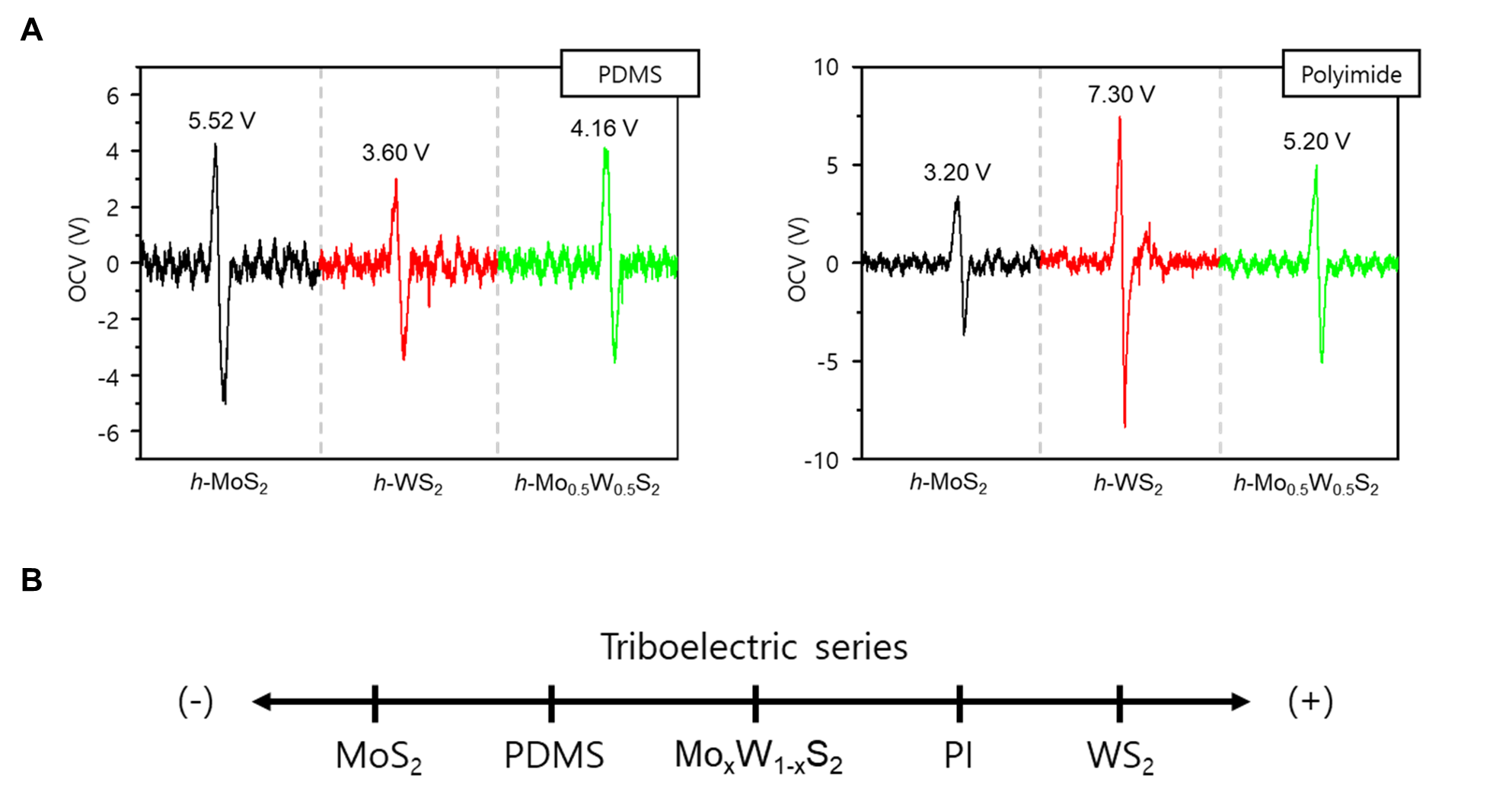


**Figure S12.** A) Measured OCVs at D_vertical_ = 1 mm using hemispherical TMDCs (*h*-MoS_2_, *h*-WS_2_, and *h*-Mo_0.5_W_0.5_S_2_) with PDMS (left) and PI (right) under humidity condition (RH: 70%). B) Measured triboelectric signals as the combination of the diversified TMDC alloys and counterpart polymeric substrate (PDMS and PI).


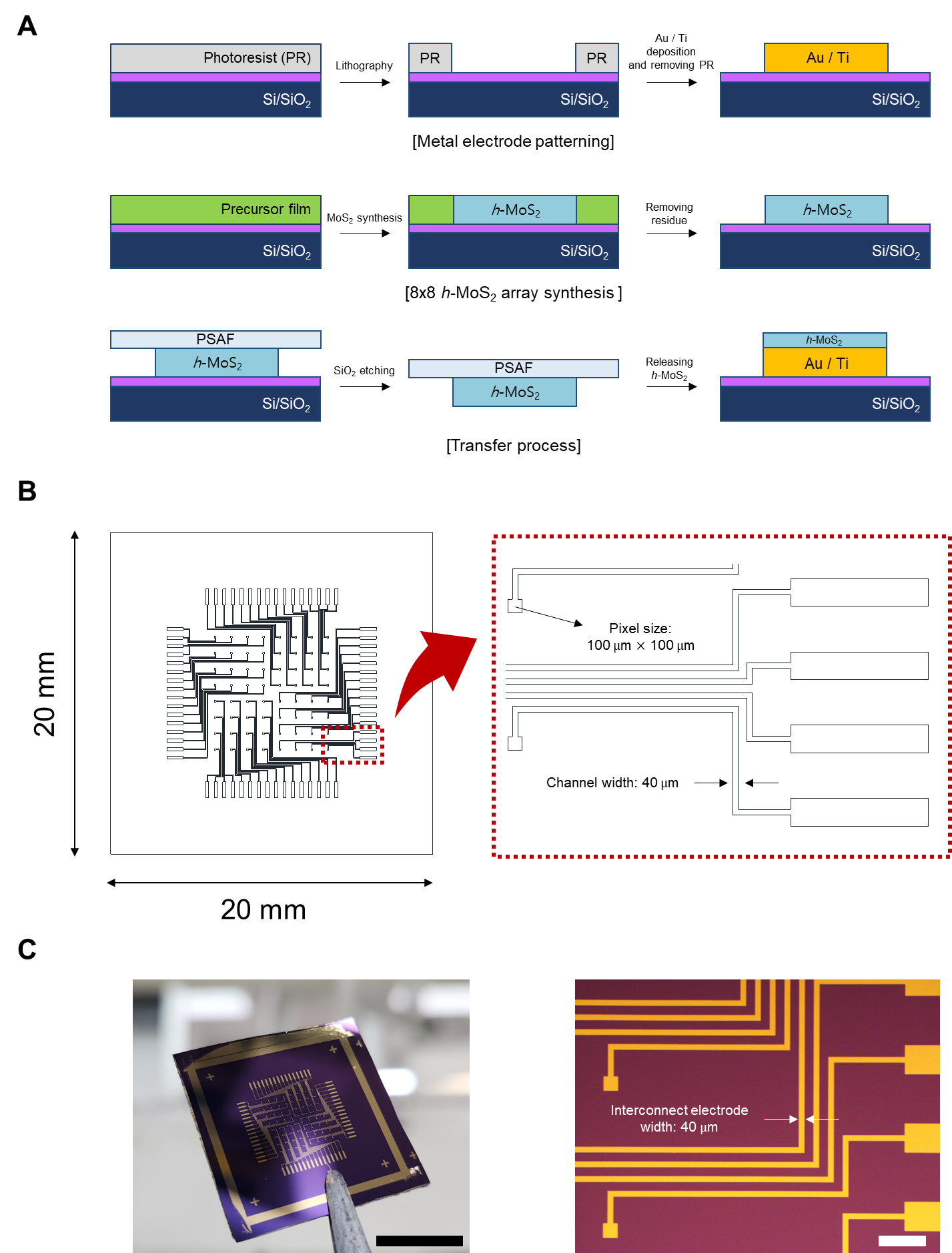


**Figure S13.** A) Schematic of the integration process for *h*-MoS_2_ and the metal electrode patterned through photolithography and area-selective annealing. B) Full photomask layout for patterned metal electrode fabrication, with a magnified view showing the individual pixel dimensions and minimum channel width. C) Photograph of the patterned electrode (scale bar: 10 mm) and a corresponding OM image (scale bar: 250 $\mu$m).

To fabricate the *h*-MoS_2_-integrated electrode, photolithography-assisted metal patterning and laser annealing were employed. A photoresist (PR) was spin-coated onto the cleaned Si/SiO_2_ substrate, followed by soft baking. Using a custom-designed photomask and UV mask aligner, the desired electrode patterns were exposed and developed to define the electrode template. Subsequently, a Au/Ti layer (50/5 nm, respectively) was deposited onto the template substrate using an electron-beam evaporator at a deposition rate of 0.1 Å/s in a high-vacuum state (<10^–6^ Torr). The patterned PR was removed by immersing the sample in a developer solution, resulting in a well-defined electrode pattern. The spin-coated MoS_2_ precursor was thermally converted into *h*-MoS_2_ pixels via selective laser annealing, and the untreated area was removed using a DMF solution (130 °C for 10 min). Subsequently, a PSAF was attached to the surface of *h*-MoS_2_ pixels, and the underlying SiO_2_ layer was etched using a prepared etchant solution consisting of hydrofluoric acid and a buffered oxide etchant in a 3:1 volume ratio. Finally, *h*-MoS_2_ pixels were cleanly transferred onto the patterned Au/Ti electrode by peeling off the PSAF.

**
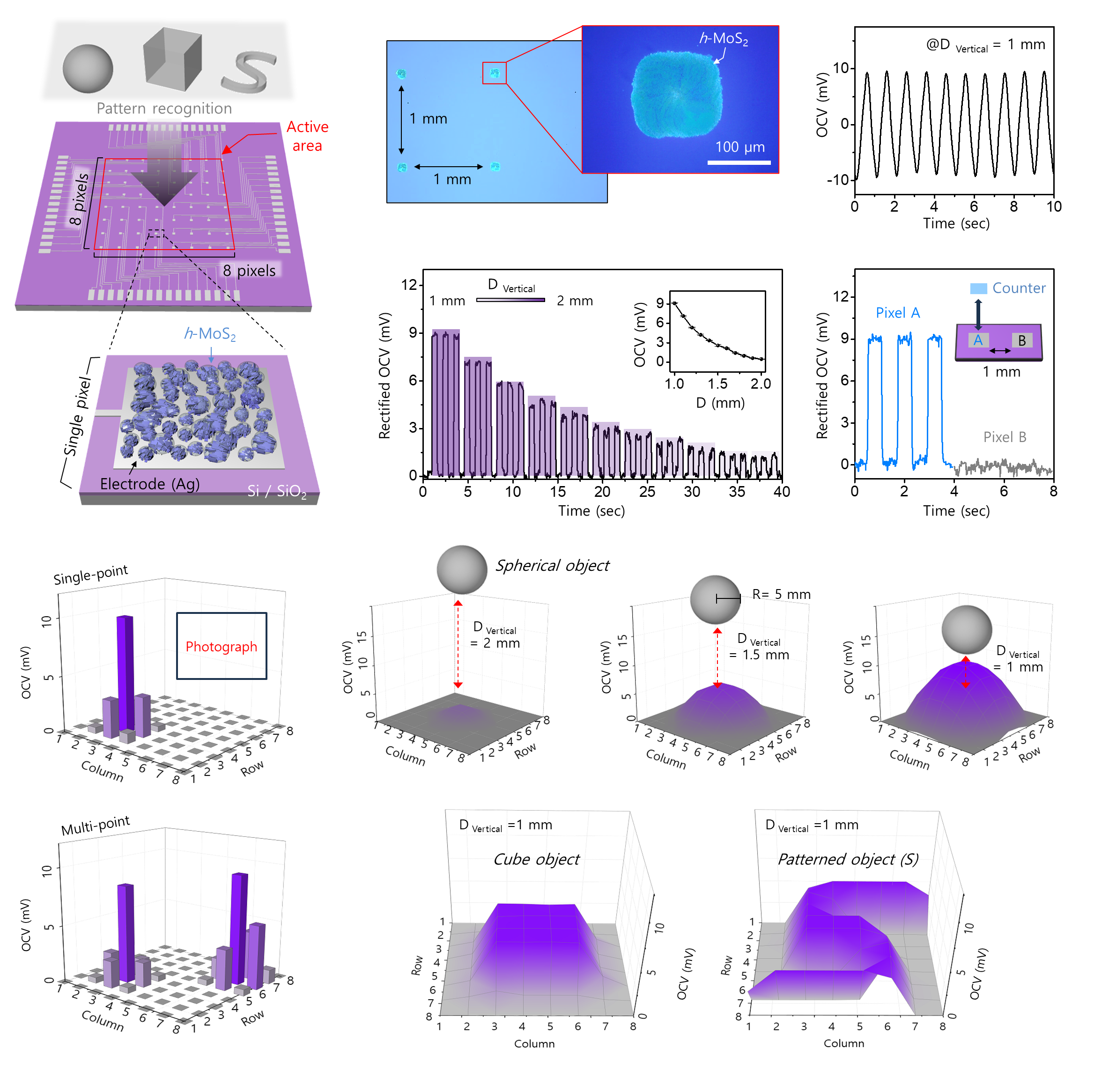
**

**Figure S14.** OM image of patterned *h*-MoS_2_ obtained via direct laser patterning synthesis with a minimum resolution of 100 $\mu$m × 100 μm.


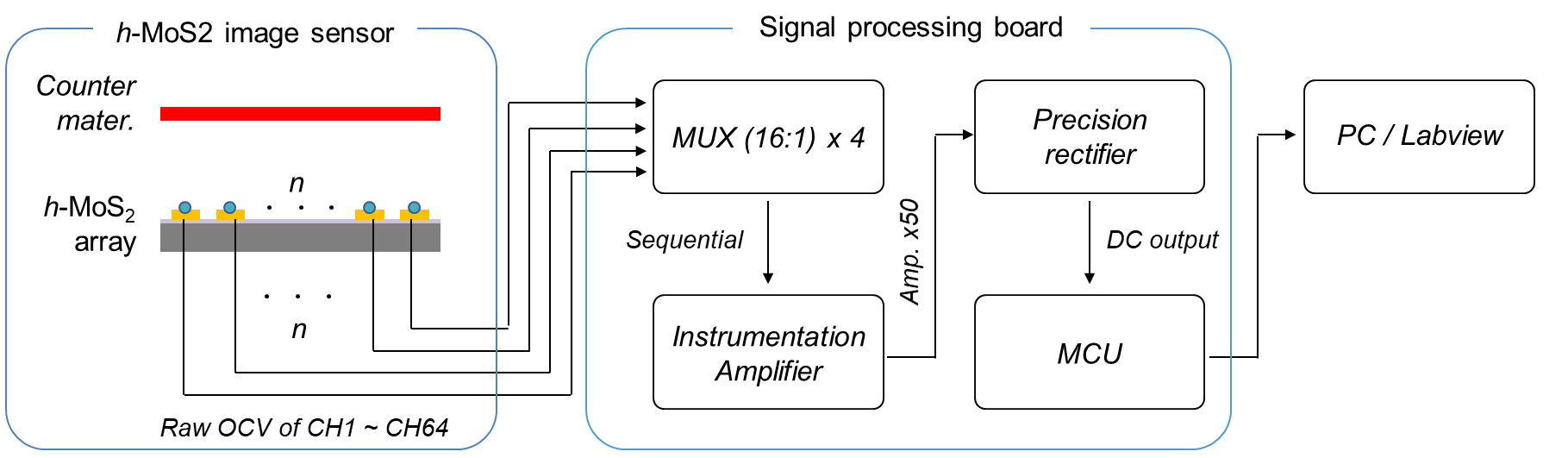


**Figure S15**. Schematic illustration of the signal acquisition and processing system for the *h*-MoS_2_ image sensor. The array consisting of 64 individual pixels generates raw open-circuit voltage (OCV) signals (CH1–CH64). These signals are sequentially selected through four 16:1 multiplexers (MUX) and then amplified by an instrumentation amplifier (gain ≈ 50×). The amplified output is converted to a direct current (DC) level by a precision rectifier, followed by digitization using a microcontroller unit (MCU). The final digital data are transferred to a PC (LabVIEW) for real-time monitoring and analysis.


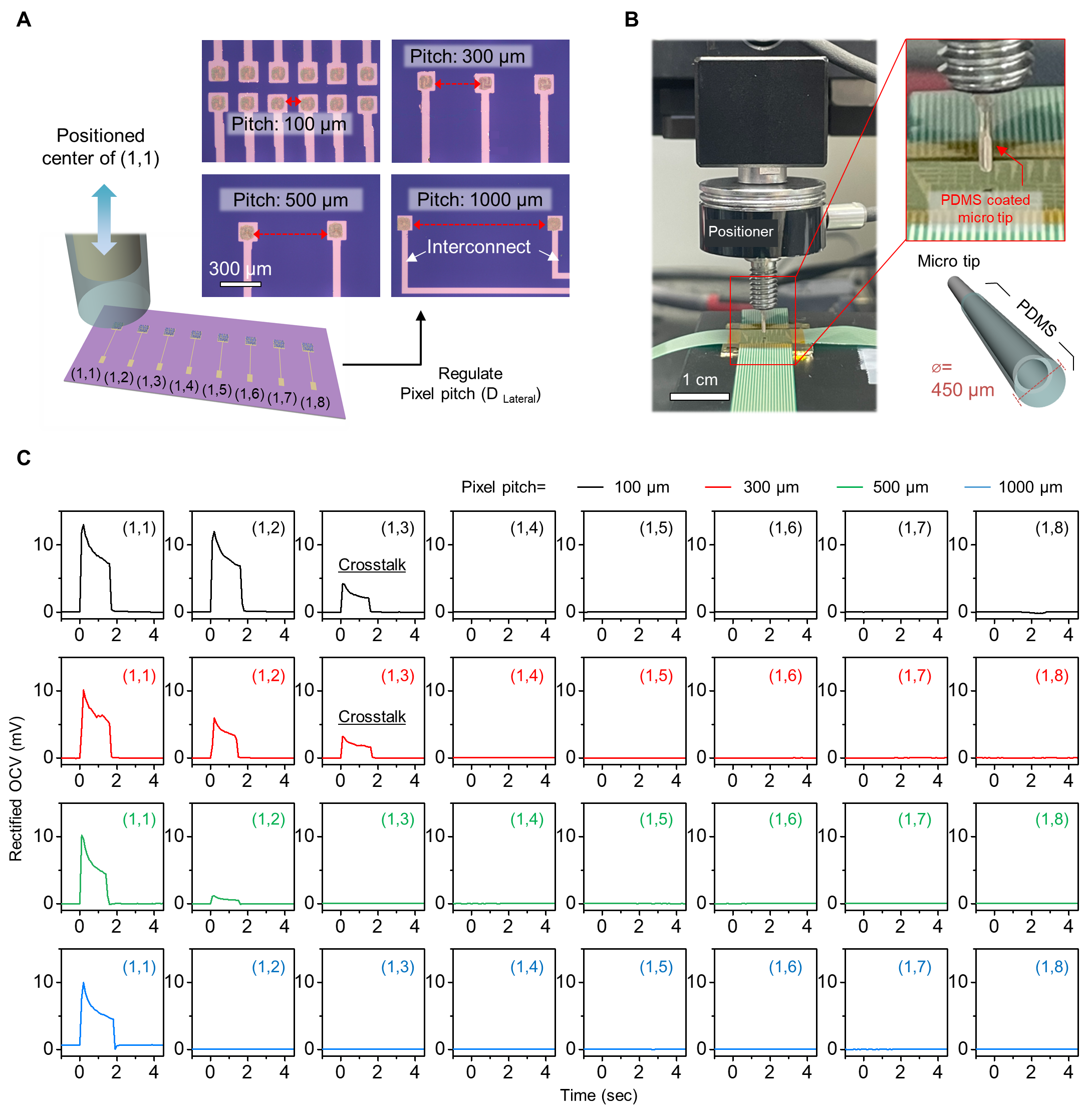


**Figure S16.** A) Schematic of the experimental setup for evaluating pixel-pitch-dependent open-circuit-voltage (OCV) crosstalk. Optical micrographs show *h*-MoS_2_ arrays with pixel pitches of 100, 300, 500, and 1000 µm. B) Photographs of the measurement setup, where crosstalk was tested using a custom PDMS-coated micro-tip (450 µm diameter). C) Rectified OCV responses of the 1 × 8 arrays, recorded while the micro-tip was vertically oscillated at the center of pixel (1,1) under each pitch condition.


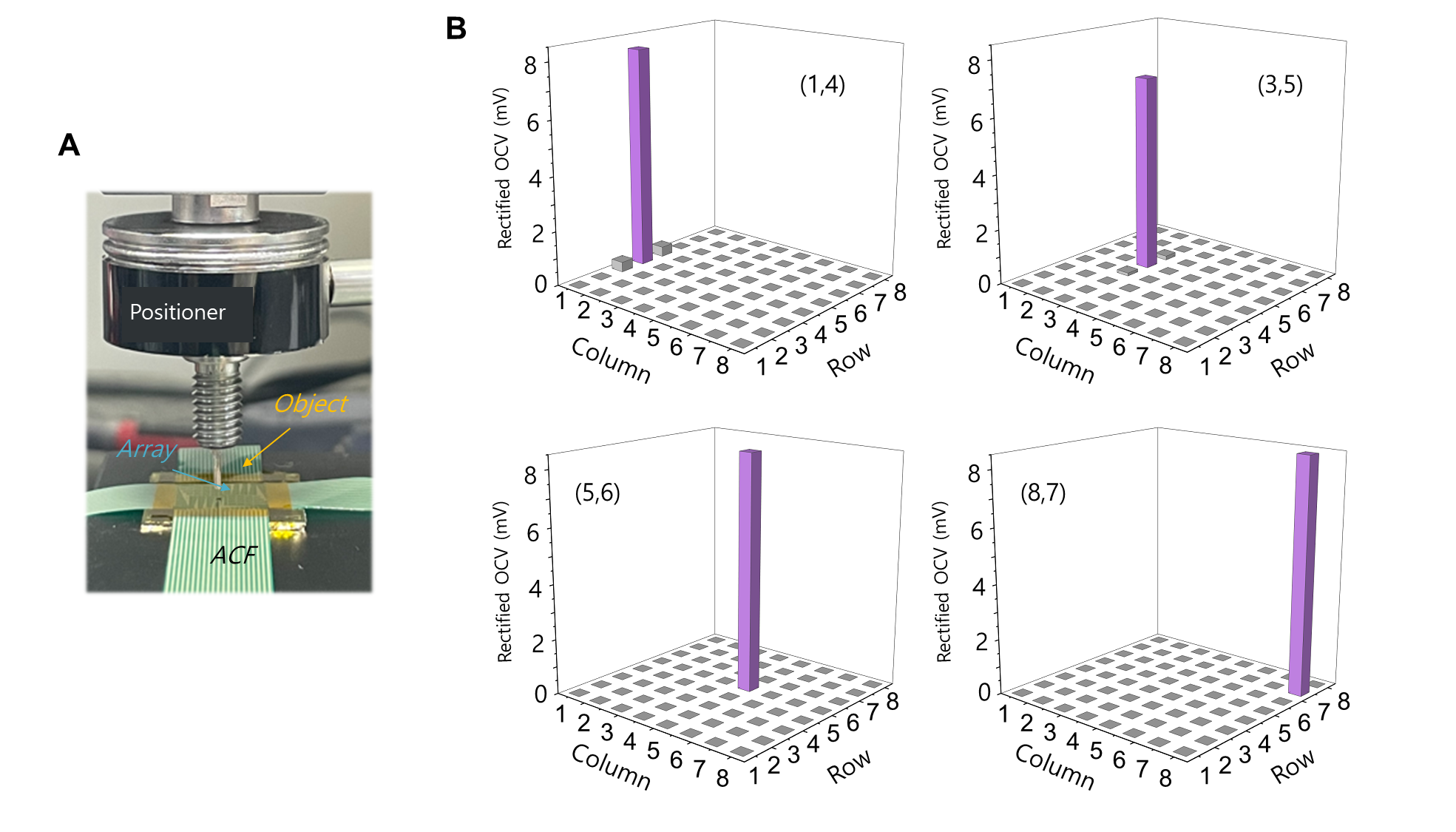


**Figure S17.** A) Photographs of the micro-scale positioning of a PDMS-coated micro-tip with a diameter of ~450 µm on the optimized 500 µm-pitch *h*-MoS_2_ array. B) Demonstration of crosstalk-free sensing performance, where the *h*-MoS_2_ image sensor reliably detects the micro-scale counter material without signal interference from adjacent pixels.


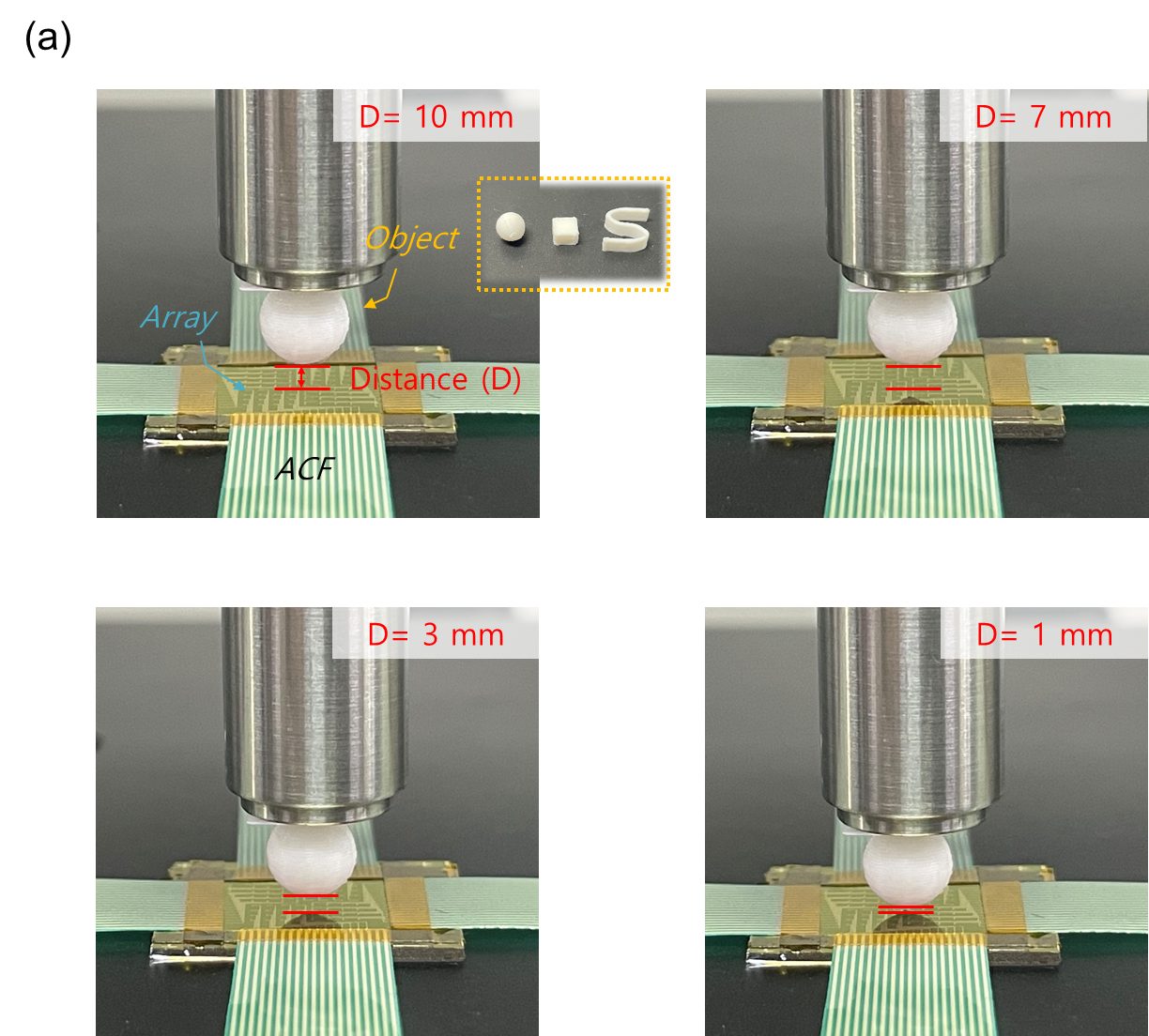


**Figure S18.** Photograph showing the measurement of OCV signals induced by 3D-printed objects at various distances (D = 10, 7, 3, and 1 mm) using an *h*-MoS_2_-based 8 × 8 pixels array connected *via* an ACF.
